# Association Between Lottery Prize Size and Self-reported Health Habits in Swedish Lottery Players

**DOI:** 10.1001/jamanetworkopen.2019.19713

**Published:** 2020-03-19

**Authors:** Robert Östling, David Cesarini, Erik Lindqvist

**Affiliations:** 1 Department of Economics, Stockholm School of Economics, Stockholm, Sweden; 2 Research Institute for Industrial Economics, Stockholm, Sweden; 3 Department of Economics, New York University, New York; 4 Swedish Institute for Social Research, Stockholm University, Stockholm, Sweden

## Abstract

**Importance:**

Poor health and unhealthy lifestyles are substantially more prevalent among individuals with low income than among individuals with high income, but the underlying mechanisms are not well understood.

**Objective:**

To evaluate whether changes to unearned wealth from lotteries are associated with long-term health behaviors and overall health.

**Design, Setting, and Participants:**

In this quasi-experimental cohort study, 4820 participants (aged 18-70 years at the time of winning) in 3 Swedish lotteries were surveyed from September 1, 2016, to November 11, 2016, between 5 and 22 years after a lottery event. Outcomes of participants in the same lottery who were randomly assigned prizes of different magnitudes by the lotteries but were ex ante identical in terms of their probability of winning different prizes were compared. Data were analyzed from December 22, 2016, to November 21, 2019.

**Exposures:**

Lottery prizes ranged from $0 for nonwinning players to $1.6 million.

**Main Outcomes and Measures:**

Four lifestyle factors (smoking, alcohol consumption, physical activity, and a healthy diet index) and 2 measures of overall health (subjective health and an index of total health derived from responses to questions about 35 health conditions).

**Results:**

The survey was returned by 3344 of 4820 individuals (69%; 1722 [51.5%] male), which corresponded to 3362 observations. The mean (SD) age was 48 (11.8) years in the year of the lottery win and 60 (11.0) years at the time of the survey. There were no statistically significant associations between prize amount won and any of the 6 long-term health outcomes. Estimated associations expressed in SD units per $100 000 won were as follows: smoking (−0.006, 95% CI, −0.038 to 0.026); alcohol consumption (0.003, 95% CI, −0.027 to 0.033); physical activity (0.001, 95% CI, −0.029 to 0.032); dietary quality (−0.007, 95% CI, −0.040 to 0.026); subjective health (0.013, 95% CI, −0.017 to 0.043); and index of total health (−0.003, 95% CI, −0.033 to 0.027).

**Conclusions and Relevance:**

In this study of Swedish lottery players, unearned wealth from random lottery prize winnings was not associated with subsequent healthy lifestyle factors or overall health. The findings suggest that large, random transfers of unearned wealth are unlikely to be associated with large, long-term changes in health habits or overall health.

## Key Points

**Question:**

Is unearned wealth from lottery winnings associated with more healthy habits and better overall health?

**Findings:**

This quasi-experimental cohort study of 3344 individuals in 3 Swedish lotteries found no statistically significant differences in long-term (5-22 years) health behaviors or overall health among individuals who participated in the same lottery but who randomly won prizes of different magnitudes.

**Meaning:**

The findings suggest that large, random transfers of unearned wealth are unlikely to be associated with large, long-term changes in health habits or overall health.

## Teaser

This quasi-experimental cohort study uses data from 3 Swedish lotteries to assess whether the magnitude of the prize won is associated with long-term health behaviors and overall health.

## Introduction

Research has documented a positive association between income and health.^1^ The positive association, sometimes characterized as the income-health gradient, has been found across a wide range of institutional settings, including developed countries with extensive welfare systems. For example, a Swedish study found that men in the lowest income decile were 5 times more likely to report being in ill health compared with men in the top decile.^2^ There is broad agreement that systematic differences in lifestyle and behavioral factors contribute to the income-health gradient.^3^ Research has consistently found that individuals with higher incomes are less likely to engage in health-impairing behaviors, such as smoking, and more likely to engage in health-promoting behaviors, such as maintaining a healthy diet or exercising regularly.^4,5,6,7^

However, to date, the reasons why health behaviors vary by income have not been established. One possibility is that income directly influences people’s health behaviors. For example, it has been hypothesized that income is associated with health behaviors because it is costly to maintain a healthy lifestyle.^8^ Some epidemiologists have also proposed that the higher prevalence of health-impairing behaviors, such as smoking, in low-income households is a behavioral response to the stress caused by limited material resources.^9,10,11^

A difficulty when testing such hypotheses is that income is rarely randomly assigned. Many estimates in the literature are therefore based on observational studies, in which concerns about confounding and reverse causality cannot be eliminated.^6^ In principle, a randomized trial could be used to study how large changes in income affect health. In practice, a well-powered randomized evaluation would likely be prohibitively expensive. Researchers are therefore increasingly turning to quasi-experimental designs when attempting to make inferences about the potential effects of income on health, but the evidence from this literature remains inconclusive.^6^

In this study, we leveraged the randomized selection of prize winners by Swedish lotteries to test whether unearned financial prizes were associated with long-term health behaviors and self-assessed health. A key methodological strength of our study that distinguishes it from previous quasi-experimental studies of survey-based measures of health^7,12,13,14,15^ is that our data allowed us to classify participants into groups within which the lottery prizes were randomly assigned via the algorithms used by each lottery. We thus compared players who had the same chance of winning the lottery but won different amounts. By using the lottery-determined randomized assignment of prize amounts within groups, we attempted to mitigate the methodological problems with observational studies mentioned above.

Our prior study^16^ of the same participants examined the association of lottery winnings with self-reported happiness, overall life satisfaction, mental health, and financial life satisfaction, finding that unearned wealth from lottery winnings was associated with improved life satisfaction but not happiness or mental health. The current study was intended to assess the associations with self-reported health behaviors.

Credible estimates of how income affects health and health behaviors are potentially valuable to policy makers. Many experts postulate that more wealth among low-income populations results in better health and healthier lifestyles.^8^ This study used the experience of lottery winners to help inform such discussions.

## Methods

### Study Design, Setting, and Participants

This quasi-experimental cohort study was approved by the regional ethical review board in Stockholm, Sweden, on April 7, 2016. The survey data were collected by Statistics Sweden in the fall of 2016. Data were analyzed from December 22, 2016, to November 21, 2019. eFigure 1 in the Supplement provides a detailed summary of the survey timeline. Informed consent was obtained in writing through survey responses or orally during telephone survey interviews. This study followed the Strengthening the Reporting of Observational Studies in Epidemiology (STROBE) guideline.

Our study builds on a previous analysis of a large administrative sample of Swedish lottery participants.^17^ That study analyzed outcomes, such as mortality and health care utilization, measured up to 10 years after the lottery event. Despite excellent power, most of the study’s quasi-experimental estimates were not statistically distinguishable from 0 (a possible exception was that large-prize winners consumed fewer anxiolytics, hypnotics, and sedatives, but the reductions were modest). A key limitation of the administrative study was that government registers do not contain information about health behaviors. As a result, the study was limited in its ability to test some of the hypotheses about the mechanisms linking income to health.

In the design stage of the present study, we identified a subset of the population in the original administrative sample. We surveyed members of the resulting survey population (N = 4820) about their overall health and health behaviors (eTable 1 in the Supplement gives additional data on how respondents were selected). The majority of the survey population received and returned the survey via regular mail. Statistics Sweden contacted a subsample of nonrespondents (n = 501) via telephone and asked them to complete an abbreviated version of the survey. The survey population was composed of participants in 3 different lotteries. One group consisted of participants in Kombi, a monthly subscription lottery with approximately 500 000 subscribers. The other 2 lottery groups consisted of winners from televised draws in a popular scratch-off lottery called Triss. We distinguished between individuals who won a monthly income supplement (Triss-monthly) and those who won a single, lump-sum prize (Triss–lump sum). For comparability, we used the net present value of the Triss-monthly installments throughout the analysis. The survey population was an approximately 1% subsample of the pooled lottery sample analyzed in the previous administrative study.^17^ However, because we oversampled large-prize winners, our statistical power compared favorably with the previous quasi-experimental studies of lottery participants.

To mitigate concerns about experimenter demand effects, the invitation letter accompanying the survey did not mention that all respondents had been drawn from lottery samples. The final survey attained an overall response rate of 69% (3344 of 4820 participants; 3233 mail respondents and 111 telephone respondents). Hereafter, we refer to the survey respondents as the respondent sample.

We publicly archived an analysis plan before accessing the survey data.^18^ The analysis plan fully specified criteria for inclusion in the estimation sample, 3 diagnostic tests of endogenous attrition, a set of primary outcomes, variable coding (including handling of missing values and outliers), the analytic framework and estimating equation, heterogeneity and robustness analyses, and procedures for multiple-hypothesis adjustment of *P* values.

### Constructing Group Identifiers

We classified participants into groups within which the prize amount was randomly assigned by the lotteries. In our analyses, we controlled for group-identifier fixed effects, thus ensuring that all estimates were derived from within-group associations between health outcomes and lottery winnings.

To construct the group identifiers, we followed previously published procedures.^17^ In Kombi, we defined a unique group identifier for each large-prize winner. We then assigned 4 nonwinners to each large-prize winner’s group. These control individuals were randomly sampled (using a preprogrammed routine in Stata [StataCorp LLC]) from the group of participants who were identical to the winners in terms of age, sex, and number of tickets purchased in the month of the win. eFigure 2 in the Supplement provides the construction of the Kombi group identifiers.

For the 2 Triss lotteries, we did not have information about lottery ticket purchases. Instead, we compared individuals who participated in large-stake televised lottery draws. Individuals qualified for the television show by winning either the lump-sum or the monthly first-stage lottery. At the show, they drew their second-stage prize from a distribution specified by a prize plan. Two individuals who qualified through the same first-stage lottery under the same prize plan faced exactly the same distribution of lottery prizes in the second stage. We therefore assigned lottery winners to the same group identifier if they won the same type of prize (lump-sum or monthly) within the same year and within the same prize plan.

### Outcome Measures

**Figure 1.**
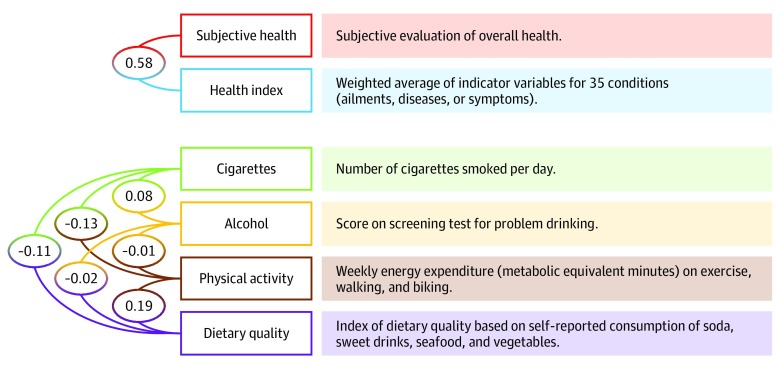
Pairwise Correlations Between Variables The numbers in the circles are the pairwise correlations (Pearson *r*) between the 2 variables connected by the lines.

Figure 1 summarizes the 6 primary outcomes (subjective health, health index, smoking, alcohol consumption, physical activity, and healthy diet) and reports their Pearson pairwise correlations (eMethods and eTable 7 in the Supplement). In selecting outcome variables, we prioritized variables that are not captured reliably by administrative registers. Four of our primary outcomes were lifestyle factors (smoking, alcohol consumption, physical activity, and healthy diet). Smoking was measured as the number of cigarettes the respondent reported smoking on a typical day. The variable was set to 0 for nonsmokers. Alcohol consumption was measured as the respondent’s score on a 3-item screening test for heavy drinking and alcohol dependence.^19^ This variable ranged from 0 to 12, with higher values indicating greater risk. Physical activity was given as an estimate of weekly energy expenditure (measured in metabolic equivalent minutes) in a typical week because of physical activity. Healthy diet was based on an index derived from responses to questions about self-reported consumption of sweet drinks, seafood, and vegetables. Health behaviors are prominently featured in both epidemiologic and economic theories of the association between income and health.^3,11,20^ Knowledge about how lottery wins are associated with health behaviors is therefore valuable for evaluating some of the mechanisms hypothesized to contribute to the income gradients.

Our final 2 outcomes were measures of overall health. Subjective health was derived from the respondent’s answer to the question, “How do you judge your overall state of health?” The variable had 5 values, ranging from a value of 1 (very poor) to 5 (very good). The other outcome, the health index, was defined as a weighted average of 35 health conditions listed in our survey. It was coded so that greater values denoted better health.

Survey-based measures of overall health are known to substantially improve the assessment of mortality risk even in specifications with a rich set of covariates.^21^ Thus, the 2 outcomes were likely to capture some information not contained in the register variables analyzed in the administrative analysis.^17^ Including these 2 variables also facilitated comparisons with the quasi-experimental estimates reported in 2 previous studies.^12,15^

### Testing Random Assignment

Previous analyses^17,22^ of the administrative sample of lottery participants from which we selected our survey population have found support for the key identifying assumption that lottery prizes are randomly assigned conditional on group identifiers. Because the data used in the previous studies were from administrative records, the tests of randomization were conducted in virtually attrition-free samples. A concern specific to the current study was that the size of the lottery prize won could be associated with survey participation. If so, our identifying assumption could fail in the respondent sample even though it held in the survey population.

To test for possible selection biases among survey respondents, our analysis began with 3 preregistered tests. In test 1, we found no evidence that survey participation was associated with the size of the lottery prize won (eTable 8 in the Supplement). In test 2, we found no evidence of covariate imbalance across groups of individuals who won prizes of different magnitudes (eTable 9 in the Supplement). In test 3, we used administrative outcomes available for all members of the survey population, including those who declined to participate in the survey. For these outcomes, the quasi-experimental lottery estimates did not change systematically when nonrespondents were omitted from the estimation sample (eTable 10 in the Supplement). Overall, the results from these tests supported the credibility of our quasi-experimental estimates.

### Additional Analyses

To provide some additional context for our lottery estimates, we compared them with income gradients and quasi-experimental studies of lottery winners’ health. Each outcome’s income gradient was defined as its association with permanent annual income (defined as net annual household income averaged over several years) in the respondent sample. To gauge the external validity of the respondent sample gradients, we compared them with gradients estimated using data on Swedish respondents in the European Social Survey.^23^ We also conducted a systematic comparison of our estimates with those in previous survey-based studies of lottery winners’ health behaviors or overall health that examined at least 1 of the outcomes in our study.^7,12,13,15^ Additional details about methods used in these comparisons are provided in the eMethods in the Supplement.

### Statistical Analysis

We used ordinary least squares (OLS) to estimate the parameters of the following regression equation: *Y_i_* = α × *P_i_* + *Z_i_* × γ + *X_i_* × β + ε*_i_*, where *Y_i_* was one of respondent *i*’s outcomes measured in SD units of the underlying variable, *P_i_* was a continuous variable for the prize amount (measured in 100 000 USD) awarded to individual *i* (thus 0 for nonwinners), *Z_i_* was a vector of control variables measured in the year before the lottery, and *X_i_* was a vector of indicator variables for the group identifiers defined above. Because we included *X_i_* in the regression, we only used within-group comparisons to estimate the lottery winnings parameter α. We used all 3 lotteries when estimating the regression, thus combining the within-group comparisons of nonwinners and winners in Kombi and the corresponding comparisons of winners of prizes of different magnitudes in the 2 Triss lotteries. The baseline controls in the vector *Z_i_* (listed in eTable 3 in the Supplement) were prespecified and were included to improve the precision of the estimated parameter α.

We used 2-sided statistical tests and a 5% significance level. All statistical analyses were performed using Stata, version 16.0, and 95% confidence intervals were calculated based on heteroskedasticity-robust SEs clustered at the level of the individual.

## Results

### Representativeness

The survey was returned by 3344 of 4820 individuals (69%; 1722 [51.5%] male), which corresponded to 3362 observations (Table 1 and eTable 2 in the Supplement). The number of observations exceeded the number of individuals because a few individuals participated in more than 1 lottery or won the same lottery multiple times. A total of 3233 responded to the mail-in survey and 111 to the abbreviated phone survey. The mean (SD) age was 48 (11.8) years in the year of the lottery win and 60 (11.0) years at the time of the survey. eTable 4 in the Supplement reports descriptive statistics for the baseline controls in the respondent sample (overall and by lottery) and the survey population.

**Table 1.** Description of Survey Population and Respondent Sample^a^

Variable	No. (%)		
	Triss–lump sum (n = 3065)	Triss-monthly (n = 570)	Kombi (n = 1205)
Prize distribution, 2011 US$			
0, Nonwinners	0	0	964 (80.0)
<10 000	811 (26.5)	0	0
10 000-100 000	2107 (68.7)	0	0
100 000-400 000	85 (2.8)	343 (60.2)	234 (19.4)
>400 000	62 (2.0)	227 (39.8)	7 (0.6)
Participation rate^b^	2055 (67.0)	378 (66.3)	929 (77.1)

^a^ Triss–lump sum was from 1994 to 2011; Triss-monthly, 1997 to 2011; and Kombi, 1998 to 2011. eTable 2 in the Supplement gives more details on the prize distribution.

^b^ Total of 3251 responded to the mail-in survey and 111 to the abbreviated phone survey.

Table 1 shows the period for which we had lottery data, the survey response rate, and the distribution of prizes awarded for each lottery (prizes in SEK net of taxes converted to 2011 USD). The survey outcomes were, on average, measured approximately 10 years (range, 5-22 years) after the lottery draw. Although most Kombi winners won prizes of approximately around $150 000, the range of prizes was greater for Triss–lump sum ($7000 to just over $900 000) and Triss-monthly ($160 000 to almost $1.6 million).

Although our quasi-experimental method did not rely on comparisons of lottery participants with nonparticipants, comparisons of lottery participants with a representative sample was used to assess whether our results were likely to generalize to the general population. eTable 4 in the Supplement provides the descriptive statistics for a representative sample of Swedish individuals from 2010 (reweighted to match the sex and age distribution of the respondent sample). In terms of educational attainment, marital status, and other baseline characteristics, all measured before the lottery events, the differences were modest. For example, 26% of the weighted survey population completed college compared with 30% in the representative sample.

We also compared the health characteristics of members of the respondent sample with those of the general population (eTable 5 and eTable 6 in the Supplement). These comparisons were subject to 2 caveats: (1) all health variables in the respondents sample were derived from survey responses obtained after the lottery, and (2) the survey questions were not always phrased identically in the 2 samples. Overall, lottery participants’ health was worse than that of the general population sample, although the differences were mostly modest. For example, 69% of lottery participants (2302 of 3338 observations) indicated that they were in good health (compared with 68% in a representative survey), 363 of 3213 (11%) smoked daily (vs 10%), 833 of 3224 (26%) engaged in at least 5 hours of physical activities per week (vs 34%), and 350 of 3237 (11%) reported never drinking alcohol (vs 14%).

### Primary Outcome Estimates

**Figure 2.**
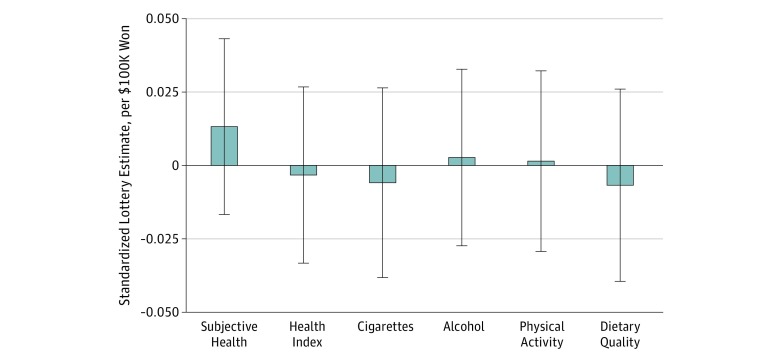
Lottery Wealth, Health Behaviors, and Overall Health Each bar represents the estimated association between lottery wealth measured in $100 000 USD (net of taxes) and outcomes measured in SD units. Error bars denote 95% CIs. Subjective health and health index are coded such that higher values denote better health. Each of the 4 behaviors is coded such that higher values denote greater prevalence of the behavior. eTable 11 in the Supplement gives underlying data and descriptive statistics.

Figure 2 gives our quasi-experimental estimates for each of the 6 primary outcomes. In all analyses, the dependent variable was measured in units of SD and lottery winnings were measured as net of taxes and in units of $100 000. All 6 estimates (reported as SD per $100 000 won) were small and not statistically significant: subjective health, 0.013 (95% CI, −0.017 to 0.043); total health index, −0.003 (95% CI, −0.033 to 0.027); smoking, −0.006 (95% CI, −0.038 to 0.026); alcohol consumption, 0.003 (95% CI, −0.027 to 0.033); physical activity, 0.001 (95% CI, −0.029 to 0.032); and dietary quality −0.007 (95% CI, −0.040 to 0.026) (Table 2 and eTable 11 in the Supplement). The 95% CIs of all estimates thus allowed us to rule out a coefficient greater than 0.05 SD units per $100 000 won.

**Table 2.** Primary Outcome Estimates

Outcome	Estimate (95% CI), SD units per $100 000 won
Subjective health	0.013 (−0.017 to 0.043)
Health index	−0.003 (−0.033 to 0.027)
Smoking	−0.006 (−0.038 to 0.026)
Alcohol consumption	0.003 (−0.027 to 0.033)
Physical activity	0.001 (−0.029 to 0.032)
Dietary quality	−0.007 (−0.040 to 0.026)

### Heterogeneity and Robustness

We reran our main analyses in subsamples stratified by sex, age at the time of the win (below or above median), prelottery income (below or above median), years since the win (before or after 2005), and type of winnings (Triss-monthly vs Triss–lump sum). The estimates were similar across subsamples, and it is unlikely that the null results reported in the full respondent sample masked strong heterogeneity (eTable 13 and eFigure 4 in the Supplement). eTable 12 and eFigure 3 in the Supplement show that our 2 preregistered robustness analyses yielded results similar to those from the primary analysis. Contrary to what one might expect if wealth has rapidly diminishing marginal effects, our coefficient estimates were broadly similar when we reran the analyses with large-prize winners ($500 000 or more) omitted.

### Benchmarking the Lottery Estimates

**Figure 3.**
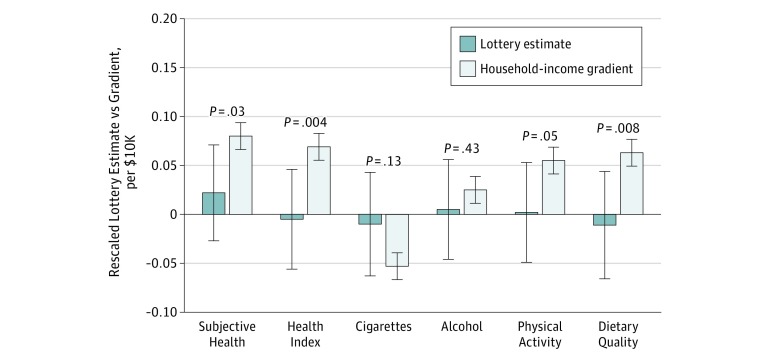
Comparison of Lottery Estimates With Household-Income Gradients Comparison between lottery estimates and household permanent income gradients. Permanent income was calculated as a multiple-year mean income in the full respondent sample. Error bars indicate 95% CIs. The *P* values are for the null hypothesis that the lottery estimate equals the gradient. Underlying estimates are given in eTable 15 in the Supplement.

Figure 3 and eTable 15 in the Supplement show the estimated gradients in the respondent sample for each of our 6 primary outcomes. We verified that the gradients in the respondent sample were similar to gradients estimated using data on Swedish respondents in the European Social Survey (eTable 14 in the Supplement). Overall, the gradients in Figure 3 replicate standard patterns in the literature, both qualitatively and quantitatively. For example, a $10 000 increase in annual household disposable income was associated with an increase in subjective health of approximately 0.080 SD units (95% CI, 0.066-0.094 SD units). Higher income was also associated with less smoking, more exercise, and better diet. In addition, we found a weak but positive association with alcohol use, which may partly reflect a higher prevalence of moderate drinking among high-income individuals.^7^ The previous literature has documented that heavy drinking is more common at lower incomes.^11^ We verified that the gradient reversed if alcohol use was redefined as an indicator variable for individuals with a score of at least 7, the cutoff for alcohol dependence recommended in a recent validation study.^24^

To compare our lottery estimates with the income gradients, we converted each lump-sum prize to an annual income by calculating the annual payout that it would generate if it were annuitized over a 20-year period. For example, a $100 000 prize would correspond to an increase in net annual income of $5996. We then compared annuity-rescaled lottery estimates with the cross-sectional gradients described above. Figure 3 shows that, for all outcomes, the rescaled lottery estimates were smaller in magnitude than the gradients and for 4 of our outcomes: subjective health, health index, physical activity, and dietary quality; the differences were statistically significant at the 5% level.

Compared with previous survey-based studies of lottery winners’ health behaviors or overall health^7,12,13,15^ that examined at least 1 of the outcomes in our study, the 95% CIs of our estimates were at least 8 times smaller. The main explanation for our substantially greater precision was that we purposefully oversampled large-prize winners. To illustrate the improved precision of our estimates, we compared our results for the health index with estimates reported in 2 previous lottery studies that analyzed comparable outcomes. One of these studies measured the index shortly after a lottery win, whereas the other analyzed a long-term health index defined similarly to ours.^12,15^ The 95% CIs in these 2 studies after rescaling to match our estimates were −0.39 to 0.35 and 0.00 to 0.50 SD units per $100 000 won, which can be compared with the 95% CI for our health index of −0.03 to 0.03. The eMethods section in the Supplement shows that the differences in precision were greater for other comparable outcomes and reports design calculations^25^ informed by our new evidence for the association between unearned wealth from lottery winnings and subsequent health.

## Discussion

**Figure 4.**
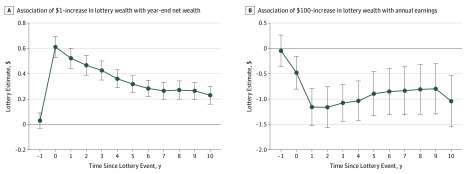
Association Between Lottery Winnings, Year-end Net Wealth, and Annual Pretax Labor Earnings Previously reported estimates of the association between lottery wealth and year-end net wealth^17^ and annual pre-tax labor earnings^22^ are shown. A, Coefficients show the association between a $1 increase in lottery prize and year-end net wealth in US dollars. Household wealth is defined as the wealth of the winner plus, if applicable, the wealth of the spouse or cohabitating partner. The register-based measure of net wealth is of high quality, but it does not capture all sources of wealth. The coefficient estimate of 0.6 for *t* = 0 should not be interpreted to mean that winners spend 40% of the lottery prize in the year of win. B, Estimates between a $100 increase in lottery wealth and annual earnings measured in US dollars based on a previous study. Error bars indicate 95% CIs.

We found no statistically significant association between the magnitude of the lottery prize won and long-term health outcomes among Swedish lottery participants. Given the discrepancy between lottery estimates and income gradients estimated in our sample, it is natural to ask if there is something particular about winning the lottery that limits the generalizability of our findings. In particular, a common view in popular culture is that lottery winners squander their wealth.^26^ If this view were correct, our null results could simply reflect most winners having fully used their winnings by the time of the survey. However, as we have discussed elsewhere,^16^ there is little evidence that lottery winners often squander their winnings. Previous analyses of the administrative sample from which we drew the survey population have found that winners reduced their labor supply and spent their lottery winnings.^17,22^ However, these adjustments were modest in magnitude and spread evenly over a long time (Figure 4). In our previous related study,^16^ lottery winnings were associated with improvements in financial life satisfaction over a decade after the lottery event (effect size, 0.067 SD units per $100 000 won; 95% CI, 0.043-0.091 SD units per $100 000 won).^16^ Interview-based studies of lottery winners in multiple countries,^26,27,28^ including Sweden,^28^ have reached similar conclusions, with 1 study of US lottery winners concluding that “contrary to popular beliefs, winners did not engage in lavish spending sprees.”^27^ Hence, the evidence suggests that the large-prize winners in our sample had better financial circumstances for many years after the lottery, but this greater financial security was not accompanied by any detectable changes in long-term health behaviors or evaluations of overall health.

### Limitations

This study has limitations. First, our study design allowed us to isolate the association between large changes in unearned wealth from lottery winnings and long-term (5-22 years) health and health behaviors of a sample of adults. Therefore, it was not suitable for detecting associations with health behaviors that dissipate within 5 years of the lottery event. Our study should also be distinguished from previous work on the association between short-term mortality and transitory income fluctuations^29^ or access to liquidity (eg, from monthly salary payments).^30,31^ Moreover, our study was conducted among a population of adults whose health behaviors may be more ingrained and thus more difficult to change than the health behaviors of younger individuals.

Second, lottery winnings differ from many other sources of income in that lottery prizes are always positive, unearned, and usually paid in lump sums. However, Triss-monthly prizes were paid as monthly installments, allowing us to test for heterogeneity by mode of payment, and we found no evidence for such heterogeneity. To the extent that earned income and unearned wealth are differently associated with health, our estimates may be most relevant as inputs into ongoing efforts to evaluate the likely costs and benefits of policy proposals that involve large, unconditional income transfers, such as basic income programs.

Third, even though the distribution of demographic and health characteristics of the participants in our sample was similar to a representative sample of Swedish adults, lottery participants may differ along unobserved dimensions in ways that limit the generalizability of our findings.

Fourth, even though income-health gradients are robust across developed countries, the underlying mechanisms may not be the same. For example, financial resources are likely to be a more important determinant of access to health care, and ultimately health, in market-based health care systems compared with countries with universal health care, such as Sweden.

## Conclusions

In this quasi-experimental cohort study, we found no association between the magnitude of the lottery prize won and long-term health outcomes among adult Swedish lottery players. For several outcomes, the lottery estimates were significantly smaller than the corresponding income gradients estimated from observational data. A plausible reason for the small lottery estimates is that lottery prizes were randomly assigned by the lotteries, so that health status and healthy behaviors could not have affected the distribution of these winnings. Overall, our results are difficult to reconcile with the view that financial resources are needed to maintain a healthy lifestyle in rich welfare states like Sweden.

## Notes

Supplement. **eMethods.** Survey Design, Representativeness, Primary Outcomes, Statistical Inference, Survey Nonresponse and Tests of Endogenous Attrition, Robustness Analyses, Benchmarking Lottery Estimates. **eReferences.** **eTable 1.** Selecting Sample of Survey Respondents **eTable 2.** Distribution of Prizes Awarded **eTable 3.** Administrative Variables and Baseline Controls **eTable 4.** Representativeness of Survey Respondents **eTable 5.** Prevalence in Respondents Sample vs Swedish Level of Living Survey **eTable 6.** Health and Habits in Respondents Sample vs Representative Survey **eTable 7.** Summary Overview of Primary Outcomes **eTable 8.** Testing Endogenous Selection into the Respondent Sample **eTable 9.** Testing for Conditional Random Assignment of Lottery Prizes **eTable 10.** Lottery Estimates in Survey Population and Respondent Sample **eTable 11.** Subjective Health and Health Behaviors (Primary Outcomes) **eTable 12.** Robustness Analyses **eTable 13.** Heterogeneity Analyses **eTable 14.** Gradients in Respondents Sample and European Social Survey **eTable 15.** Comparison to Permanent Income Gradients **eFigure 1.** Schematic Overview of Timeline for Collection of Survey Data **eFigure 2.** Illustration of Identification Strategy in Kombi Sample **eFigure 3.** Results from Pre-registered Robustness Analyses **eFigure 4.** Results from Pre-registered Heterogeneity Analyses
