# Supplementary material for: Association Between Lottery Prize Size and Self-reported Health Habits in Swedish Lottery Players
Source: JAMA Netw Open. 2020 Mar 19;3(3):e1919713. doi: 10.1001/jamanetworkopen.2019.19713 (PMC7082720; doi:10.1001/jamanetworkopen.2019.19713)
Supplement: Supplement. — eMethods. Survey Design, Representativeness, Primary Outcomes, Statistical Inference, Survey Nonresponse and Tests of Endogenous Attrition, Robustness Analyses, Benchmarking Lottery Estimates. eReferences. eTable 1. Selecting Sample of Survey Respondents eTable 2. Distribution of Prizes Awarded eTable 3. Administrative Variables and Baseline Controls eTable 4. Representativeness of Survey Respondents eTable 5. Prevalence in Respondents Sample vs Swedish Level of Living Survey eTable 6. Health and Habits in Respondents Sample vs Representative Survey eTable 7. Summary Overview of Primary Outcomes eTable 8. Testing Endogenous Selection into the Respondent Sample eTable 9. Testing for Conditional Random Assignment of Lottery Prizes eTable 10. Lottery Estimates in Survey Population and Respondent Sample eTable 11. Subjective Health and Health Behaviors (Primary Outcomes) eTable 12. Robustness Analyses eTable 13. Heterogeneity Analyses eTable 14. Gradients in Respondents Sample and European Social Survey eTable 15. Comparison to Permanent Income Gradients eFigure 1. Schematic Overview of Timeline for Collection of Survey Data eFigure 2. Illustration of Identification Strategy in Kombi Sample eFigure 3. Results from Pre-registered Robustness Analyses eFigure 4. Results from Pre-registered Heterogeneity Analyses [file jamanetwopen-3-e1919713-s001.pdf]

Supplementary Online Content

Östling R, Cesarini D, Lindqvist E. Association between lottery prize size and self-reported health habits in Swedish lottery players. *JAMA Netw Open*. 2020;3(3):e1919713. doi:10.1001/jamanetworkopen.2019.19713

eMethods. Survey Design, Representativeness, Primary Outcomes, Statistical Inference, Survey Nonresponse and Tests of Endogenous Attrition, Robustness Analyses, Benchmarking Lottery Estimates.

eReferences.

eTable 1. Selecting Sample of Survey Respondents

eTable 2. Distribution of Prizes Awarded

eTable 3. Administrative Variables and Baseline Controls

eTable 4. Representativeness of Survey Respondents

eTable 5. Prevalence in Respondents Sample vs Swedish Level of Living Survey

eTable 6. Health and Habits in Respondents Sample vs Representative Survey

eTable 7. Summary Overview of Primary Outcomes

eTable 8. Testing Endogenous Selection into the Respondent Sample

eTable 9. Testing for Conditional Random Assignment of Lottery Prizes

eTable 10. Lottery Estimates in Survey Population and Respondent Sample

eTable 11. Subjective Health and Health Behaviors (Primary Outcomes)

eTable 12. Robustness Analyses

eTable 13. Heterogeneity Analyses

eTable 14. Gradients in Respondents Sample and European Social Survey

eTable 15. Comparison to Permanent Income Gradients

eFigure 1. Schematic Overview of Timeline for Collection of Survey Data

eFigure 2. Illustration of Identification Strategy in Kombi Sample

eFigure 3. Results From Pre-registered Robustness Analyses

eFigure 4. Results From Pre-registered Heterogeneity Analyses

This supplementary material has been provided by the authors to give readers additional information about their work.

© 2020 Östling R et al. *JAMA Network Open.*

eMethods. Survey Design, Representativeness, Primary Outcomes, Statistical Inference, Survey Nonresponse and Tests of Endogenous Attrition, Robustness Analyses, Benchmarking Lottery Estimates.

The survey data we analyze were collected on behalf of the research team by Statistics Sweden in the fall of 2016. The survey was designed to gather comprehensive information about three domains: (i) subjective health and lifestyle (ii) psychological well-being and (iii) political and moral attitudes. This study reports the results from our analyses of outcomes in the first of these domains. The companion paper on psychological well-being is publicly available in preprint form.[1]

The Analysis Plan[2] is accessible via the URL https://osf.io/t3qb5/. All major aspects of the analyses – criteria for inclusion in the estimation sample, heterogeneity analyses, robustness analyses, estimating equation used in empirical analyses, diagnostic tests for endogenous attrition, variable coding (including handling of missing values and outliers), and multiple-hypotheses adjustment – were fully specified. Here, we summarize these methods. We also describe the methods used in our comparisons of our lottery estimates to income-health gradients and quasi-experimental estimates from the literature. The Analysis Plan declared our intention to make such comparisons, but did not specify in full the procedures to be used.

Several key passages in our descriptions below are reproduced verbatim or in lightly edited form from the Analysis Plan. Since the present study and the companion study[1] rely on common and pre-registered procedures, there is substantial overlap between the two studies in the language used to describe procedures and results from pre-registered analyses that do not depend on the outcomes analyzed (e.g. the diagnostic tests of covariate balance).

Survey Design

Survey Population. The outcomes we analyze were obtained by surveying a subset of players selected from a large administrative sample of lottery players (from four separate lottery populations). The administrative lottery sample has negligible attrition and has been used in several previous studies of outcomes measured in government registers.[3–5] In determining which players to target by our survey, we sought to balance multiple objectives, but a major goal was to preserve a lot of the exogenous variation in wealth generated by the lottery prizes awarded to players in the administrative sample.

Our final *Survey Population* consists of players selected from three of the four lotteries in the administrative sample: Kombi, Triss-Monthly and Triss-Lumpsum. Kombi is a subscription lottery with approximately 500,000 subscribers, the proceeds of which are donated to the Swedish Social Democratic Party. We have data on Kombi participants between 1998 and 2011. Triss is a popular scratch-off lottery held in Sweden and there are two types of major prizes. We have data on Triss-Monthly winners from 1997 until 2011. They win a monthly income supplement, the size and duration of which are drawn randomly and independently. Winners of the Triss-Lumpsum (1994 to 2011) prizes are instead awarded a lump-sum prize, the size of which is also drawn randomly from a known distribution. We elected *not* to survey participants in the fourth lottery used in our prior studies, namely the prize-linked savings (PLS) sample because most large lottery prizes were awarded in the late 1980s and early 1990s, making is less likely to detect health differences in 2016.

To select the *Survey Population*, we began by identifying all Triss-Lumpsum winners, all Triss-Monthly winners and all large-prize winners from Kombi (defined as players who won at least 1M SEK). The final *Survey Population* was subsequently selected using the following stepwise procedure:

1. We dropped prizes if the winning player's personal identification number ("PIN") could not be reliably determined or if key covariates (e.g., information about the number of tickets owned in Kombi) were missing.
2. From each of the two Triss samples, we dropped subjects for whom we had indications that the winning ticket was jointly owned. Such prizes constitute ~7% of the sample (for details on joint ownership, see Section IV in the Online Appendix of our previous study[3]). We also dropped a small number of Triss players who won multiple prizes under the same prize plan.
3. We restricted the sample to prizes won by players aged 18 or above at the time of win and who were at most 75 years of age at year-end in 2016 (the year of the survey). The upper age limit is motivated by evidence that survey nonresponse increases with age.[6] We also dropped players deceased by 2011 (the last year for which we have data on mortality).

© 2020 Östling R et al. *JAMA Network Open.*

4. For each large-prize event in Kombi, we sought to identify suitable experimental controls. A non-winning player was deemed a suitable control if their sex, year of birth and number of tickets owned (in the month of win) were identical to that of the winner. For three large-prize winners, we were unable to identify four controls satisfying these criteria; we therefore dropped them.
5. The above restrictions left us with 259 large-prizes from Kombi, 3,294 Triss-Lumpsum prizes and 608 Triss-Monthly prizes. We supplied information about these winners to Statistics Sweden, who dropped prizes won by individuals who were deceased or lacked an official Swedish address of residence in 2016. These restrictions leave us with 241 Kombi prizes, 3,065 Triss-Lumpsum prizes and 570 Triss-Monthly winners.
6. In a final step, we added four experimental controls for each large Kombi prize.

The upper panel of eTable 1 summarizes the number of observations dropped because of each restriction. Applying all the restrictions leaves us with our *Survey Population* of 4,840 observations (4,820 unique individuals): 241 Kombi large-prize events and 964 (241×4) matched controls, 3,065 Triss-Lumpsum prizes and 570 Triss-Monthly prizes.

Survey Protocol. Having defined the *Survey Population*, Statistics Sweden began sending out mail-in surveys in the fall of 2016 (see eFigure 1 for details about the timeline of the survey). Surveyed players were initially mailed a letter of invitation accompanied by the eight-page survey, a return envelope, and a 100 SEK gift certificate included to encourage survey participation. The cover letter explained that subjects who chose to return the mail-in survey were also consenting to having their survey responses linked to administrative registers about socio-economic outcomes.

As a condition for conducting the survey, Statistics Sweden required that information about these registers be provided to interested subjects, along with information about the selection of the *Survey Population*. We did not wish to make salient to subjects why they were being surveyed, out of fear that any mention of lotteries might color their responses. Therefore, the cover letter made no reference to individual lotteries or to the administrative lottery sample from which we had identified the *Survey Population*. Subjects interested in more information about the administrative registers or the selection of the *Survey Population* were instead referred to a website. Unbeknownst to the subjects, each letter's website URL was unique, and the final data delivered to us therefore contains information about which subjects accessed the website. Only six subjects did, so any biases are likely to be negligibly small.

According to the survey protocol agreed to with Statistics Sweden, subjects who failed to return a survey after the first mailing were sent three reminder letters, the last two of which also included new paper copies of the survey. In a next step, subjects in the Triss-Monthly sample who failed to return a survey after the third reminder were also contacted by telephone and asked to return the survey. Subjects who acquiesced were mailed a new copy of the survey if required. Efforts to establish contact ceased after four calls. For budgetary reasons, we restricted the phone-call reminders to players in the Triss-Monthly sample (observations from this lottery contribute more treatment variation, on average, and are hence more valuable in terms of improving the precision of our estimates).

Respondents Sample. Three weeks after the end of the regular data-collection, Statistics Sweden conducted a follow-up study on a randomly selected subset of 501 subjects who failed to return a mail-in survey. Each subject was invited to participate in an abbreviated version of the survey via the telephone. The phone interview was designed to take six minutes. Statistics Sweden made five attempts at contact before abandoning efforts to obtain responses to the abbreviated telephone version of the survey.

In total, the survey attained a response rate of 69% (see eTable 2 for response rates by lottery). Here and in what follows, we refer to the survey participants as our *Respondents Sample*. In eTable 2, we show the distribution of lottery prizes overall and by lottery, in both the *Survey Population* and the *Respondents Sample.* All prize amounts are net of taxes and measured in units of year-2011 dollars. For comparability, the Triss-Monthly prizes are converted to a net-present-value amount. Even though the *Survey Population* is approximately a 1% subsample of the "pooled lottery sample" analyzed in our previous study,[3] the oversampling of large-prize winners allowed us to retain about half of the identifying variation in the original administrative sample.

In addition to the survey data, the data set Statistics Sweden ultimately delivered to us also contains a number of demographic variables from administrative registers and some lottery-specific variables needed to construct the group identifiers. The administrative variables are shown in Panel A of eTable 3 and are available also for survey nonrespondents. Panel B instead reports a set of baseline characteristics used throughout several analyses.

To reduce concerns about investigator degrees of freedom in the selection of our estimation sample, we also defined the procedures by which we would select our estimation sample in each analysis. Following the Analysis Plan, all our analyses are conducted in the largest attainable sample of individuals with non-missing outcome data

© 2020 Östling R et al. *JAMA Network Open.*

and baseline characteristics in the year prior to the lottery. (In the Analysis Plan, we noted that under some scenarios, the three diagnostic tests described below could produce results that may justify ex-post changes to the sample-selection criteria, but we committed a priori to clearly describe any such revisions as departures from the original strategy in the eventually published study. Fortunately, no such revisions were deemed necessary and all analyses in the main text were conducted in estimation samples constructed exactly following our pre-registered procedures.)

Representativeness

eTable 4 compares the distributions of demographic characteristics of players in the *Respondents Sample*, overall as well as by lottery, the *Survey Population* and a representative sample that has been reweighted to match the sex and age distribution of the *Respondents Sample*. The demographic characteristics are the baseline characteristics defined in eTable 3, albeit with age and age squared replaced by year of birth. The baseline characteristics of lottery players are measured at year-end in the year prior to the lottery and are similarly distributed in the *Respondents Sample* and the *Survey Population*. Column (6) also shows the baseline characteristics for a random sample of Swedish adults drawn in 2010 after it has been reweighted to match the sex and age distribution of the *Respondents Sample*. Columns (4) and (6) suggest that in terms of these observable baseline characteristics, the two samples are similar. A previous analysis[3] of the administrative sample from which the *Survey Population* was drawn also found that, adjusting for compositional sex- and age differences, the health-care utilization and mortality of lottery players resembles that of the Swedish population as a whole.

In additional analyses, we calculated the prevalence of 35 ailments, diseases and health conditions included in our survey and compared the results to a representative sample of Swedes. (Further information about the 35 conditions is available in the description of our *Health Index* below.) eTable 5 reports the prevalence of each condition in the *Respondents Sample* and the 2010 wave of the Swedish Level of Living Survey (SLLS). The SLLS numbers are calculated in a sample that has been reweighted to match the sex- and age distribution of the *Respondents Sample*. The comparison is subject to some interpretational caveats. First, the questions used in our survey were not formulated identically to those used by the SLLS. Second, the SLLS questions offered several response alternatives designed to measure the intensity of any symptoms, whereas our survey questions did not. Third, the SLLS data are based on face-to-face interviews conducted in 2010, whereas our data are based on a mail-in-survey administered in 2016. Prevalence is greater in the *Respondents Sample* for 22 out of 35 conditions and smaller for the remaining 13 conditions. In most cases, the differences are small in magnitude, however.

To further assess representativeness, we used publicly available data from the 2016 wave of the Swedish Public Health Survey, a representative survey whose methodology is similar to ours. We compared the prevalence of nine indicator variables constructed from questions that were identical across the two surveys. In these analyses, we continue reweight the data from the representative sample to match the sex- and age distribution of our *Respondents Sample*. The publicly available summary statistics only contain data for four sex- and age categories, so the reweighting is unfortunately coarser than in other analyses. eTable 6 suggests that members of the *Respondents Sample* have somewhat worse health and health habits overall.

Primary Outcomes

The primary outcomes and their pairwise correlations are summarized in eTable 7. Our six primary outcomes were defined as follows.

Subjective Health. This variable is based on the subject's response to the question "How do you judge your overall state of health?". Subjects are offered five response categories, ranging from "Very Poor" to "Excellent". We assigned a numerical value of 1 for subjects who selected "Very Poor", 2 for "Poor" and so on up to 5 for "Excellent".

Health Index. Our survey contains a question adapted from the SLLS.[7] The original survey lists 51 health conditions (an ailment, disease or symptom) and asks the respondent to indicate whether or not they have suffered from each of the conditions in the past 12 months (and if yes, how much). To economize on survey space, we excluded the 16 conditions with the smallest pairwise correlations with subjective health. We asked subjects to indicate whether they suffered from each of the conditions, but not about the severity of any symptoms.

The 35 health conditions we retained in the survey are listed in Table S5. In addition to these 35 categories, the survey question contains an additional response: "None of the above ailments or diseases during the past twelve months". Respondents who did not check any of the boxes (including the "None of the above…." option) are treated as missing in our analyses. The health index variable, whose construction is described below, is defined

© 2020 Östling R et al. *JAMA Network Open.*

for all other respondents. (Table VI in the Analysis Plan incorrectly listed genital discomfort as one of the 35 conditions covered by our survey and inadvertently omitted nausea.).

Our procedure for aggregating the 35 conditions into a health index is similar to that used by Lindahl.[8] Specifically, we regress *Subjective Health* on indicator variables for each of the 35 conditions, a cubic in age, sex, and sex-by-age interactions. We restrict the estimation sample to individuals aged 18-75 at the time of the survey. We then use the coefficients estimated from SLLS to predict each respondent's subjective health from their covariates $\boldsymbol{X}_i$. Our final index is simply this predicted value after it has been standardized to have mean zero and unit variance in our estimation sample.

Smoking. Our survey asks respondents to state whether they smoke daily, occasionally or never. Daily smokers are also asked how many cigarettes they smoke per day. Our primary outcome is the number of cigarettes the respondent smokes per day (responses were restricted to be a positive integer below 100). For daily smokers, we set this variable equal to the number of cigarettes smoked per day, censoring the variable at 60, a threshold corresponding to three packs of cigarettes per day. For occasional smokers, we set the variable equal to 1 cigarette per day (thus ensuring that no occasional smokers has a recorded smoking quantity strictly greater than the minimum quantity a daily smoker can report). For never smokers, we set the variable's value equal to 0.

Alcohol. This variable is the respondent's score on a 3-item screening test for problem drinking. We use a previously described procedure to determine each respondents score.[9] The first question asks respondents how often they had an alcoholic beverage last year. Possible answers are never (0 points), monthly or less (1 point), 2 to 4 times a month (2 points), 2 to 3 times a week (3 points) or 4 or more times a week (4 points). The second question asks respondents how many drinks they consumed on a typical drinking day during the past year, and respondents were showed a picture of what a standardized drink refers to. Responses are coded as followed: 1 to 2 drinks (0 points), 3 to 4 drinks (1 point), 5 to 6 drinks (2 points), 7 to 9 drinks (3 points), or 10 or more drinks (4 points). The third question asks how often the respondent had 6 or more drinks at one occasion during the last 12 months. Responses were less than monthly (1 point); monthly (2 points), weekly (3 points), or daily or almost daily (4 points). With one exception, the primary outcome measure is the sum of points from the three questions, i.e. a possible score between 0 and 12. The exception is that individuals who answer "never" to the first question are scored as zero (regardless of their responses to the remaining questions). Respondents whose response to the first question indicates some drinking are coded as missing unless they respond to both follow-up questions.

Physical Activity. Our survey contains two questions about physical activity inspired by the International Physical Activity Questionnaire (IPAQ). The short IPAQ questionnaire asks respondents to indicate how many minutes per week they spend on nine separate activities.[10] Physical activities are weighted by their metabolic equivalent (MET) to form an overall measure of the total number of MET-minutes per week. Our first question asks how much time the respondent spends exercising during a regular week and the second how much is spent doing moderately physically demanding everyday activities like walking and biking. We assume that MET for the activities in the first and second question are 8 and 4, respectively. To make it easier for respondents to answer the questions, we did not allow open-ended answers and provided a number of response alternatives, e.g. 0, 0-29, 30-59, 60-89, 90-119, 120 minutes or more for the question about exercise. We translate each response into minutes using the midpoint of these intervals. For the highest choice alternative, we add half the distance between the second highest choice alternative, i.e. 120 + 15 minutes for exercise. If a respondent has only answered one of the two questions, it is coded as missing. The resulting measure of MET-minutes per week is our primary outcome.

Healthy Diet. This variable is derived from responses to three questions about dietary habits. The first question asks respondents how often they eat vegetables (excluding potatoes). The second asks how often they consume soda and other sweet drinks and the last question how often they eat seafood. Each question has between 5 and 7 response alternatives. For the questions about seafood and vegetables, we assign the number 0 to the response indicating the lowest frequency of consumption, 1 to the response indicating the second lowest frequency, and so on. For soda, we proceed the same way, but reverse-code the responses so that higher values denote lower consumption of soda. We subsequently standardize the three numerical variables. Our final index is the sum of the three (standardized) variables. For subjects with exactly one missing item-level response, we replace the missing value by the question-specific mean before calculating the value of the index. We set the index to missing if at least two of the questions have missing values.

Statistical Inference

Throughout, we report *p*-values based on analytical standard errors that have been clustered[11] at the individual level. In our main analysis of the primary outcomes, we also report permutation-based *p*-values constructed by simulating the distribution of the coefficient estimates under the null hypothesis of no association. In each

© 2020 Östling R et al. *JAMA Network Open.*

simulation iteration, we independently permute the prize column in each group. We next use our estimating equation to generate an estimate of α and its standard error. Repeating this process 10,000 times gives us a simulated distribution that we use to calculate the probability of observing a test statistic as extreme as the one observed under the null hypothesis. Finally, in our main analyses of the primary outcomes, we also report *p*-values that have been adjusted to account for the fact that we examined six primary outcomes. To calculate these family-wise error rate adjusted *p*-values, we apply the free step-down resampling method of Westfall and Young.[12] In the tables, we refer to the resulting *p*-values as FWER-adjusted *p*-values.

Survey Nonresponse and Tests of Endogenous Attrition

A potentially serious concern is the possibility that the lottery outcome is related to the likelihood that a respondent agrees to participate in the survey. Such endogeneity can lead to violations of the key identifying assumption for causal inference (i.e., that the treatment is independent of potential outcomes conditional on our group-identifier effects) in the *Respondents Sample* even if it holds in the *Survey Population*. To test for selection biases, we pre-specified three distinct tests for endogenous selection. Below, we present these tests and the results.

Diagnostic Test 1. Association between Wealth and Survey Participation. In our first diagnostic test, we examined whether survey participation was associated with prize amount. The results from this test are shown in eTable 8. The first two columns report coefficient estimates from a regression of an indicator variable equal to 1 for subjects who returned a mail-in survey and 0 for subjects who did not, on prize amount won. The results without group identifier fixed effects are shown in column 1 and the results with the group identifier fixed effects are in column 2. Column 3 shows the results from an analogous specification estimated among players invited to the abbreviated telephone survey. Here, the dependent variable is an indicator equal to one for subjects who agreed to participate. Finally, column 4 shows the results from a specification in which survey participation is defined as either having returned the mail-in survey or having answered the abbreviated telephone survey. Across all specifications, we fail to see any indications that lottery prize is associated with survey participation.

Diagnostic Test 2. Testing for Balance in Baseline Characteristics. Our second pre-specified test checks for covariate imbalance using the following estimating equation:

$$P_i = \boldsymbol{X}_i \times \boldsymbol{\beta} + \boldsymbol{Z}_i \times \boldsymbol{\gamma} + \varepsilon_i, \qquad (2)$$

where P_i is the prize amount, $\boldsymbol{X}_i$ are indicator variables for the group identifiers and $\boldsymbol{Z}_i$ the pre-specified baseline characteristics measured in year prior to the lottery event. The Analysis Plan showed that the covariates are balanced in the *Survey Population*, as indeed one should expect given that it is virtually attrition free. In eTable 9, we reproduce the results from the original analyses of the *Survey Population*, alongside results from analogous analyses conducted in the *Respondents Sample*. Controlling for the group-identifier fixed effects, no individual coefficient estimate is statistically distinguishable from zero at the 5% level and an *F*-test of the joint significance of the baseline characteristics also fails to reject the null hypothesis that pre-lottery characteristics are jointly predictive of the lottery outcome. These conclusions hold in both the *Survey Population* and the *Respondents Sample*. We reach similar conclusions when Kombi is analyzed separately from the two Triss lotteries. (The Kombi specification omits sex and age, because these variables never vary among players in the same group.)

Diagnostic Test 3. Lottery Estimates in Survey Population vs Respondents Sample. In our third test, we estimated the association between lottery wealth and a number of register-based outcome variables in the *Survey Population* and examined whether the coefficients moved appreciably when the estimation sample was restricted to the *Respondents Sample*. Evidence of systematic differences between the two sets of coefficient estimates could, but need not, be an indication of endogenous selection into the *Respondents Sample*. In eTable 10, we report the results from these analyses. In columns 1, 3, 5, and 7 we report estimates from the *Survey Population* (the smaller sample sizes in columns 1 and 3 reflect the fact that financial variables are only available 2000-2007 and net wealth and debt at year-end in the year of the lottery event is only defined for players who won in these years). In columns 2, 4, 6, and 8, we report the results from exactly analogous analyses conducted with non-respondents omitted from the estimation sample. For all pre-specified outcomes – t = 0 net wealth, t = 0 debt, t = 1 capital income, and t = 1 labor income – the estimates are similar in magnitude.

Robustness Analyses

Our Analysis Plan also specified a set of robustness analyses, the results of which are reported in eTable 12 and eFigure 3. In our first robustness analysis, we omitted large-prizes winners. Our original Analysis Plan defined prizes above 4M SEK (year-2011 prices) as large. For expositional ease, we rounded this cutoff to 500K USD (year-2011 prices) in eFigure 3. For completeness, eTable 12 reports results for both cutoffs, and unsurprisingly,

the results are highly similar. Dropping large prizes increases standard errors, but the coefficient estimates are broadly similar, suggesting that our main findings are unlikely to be driven entirely by large wealth shocks. In our second robustness analysis, we reran the main analysis for *Subjective Health* weighting mail-in survey respondents and telephone survey respondents to match the population response rate to the mail-in survey. The reweighting leads to a somewhat larger estimate.

Benchmarking Lottery Estimates

Rescaling Lottery Estimates for Comparability with Gradients. Since much of the literature on the relationship between income and health is correlational, we expected that some readers would find it informative to compare our lottery estimates to income-health gradients. In this section, we therefore describe and motivate the methodology used to generate the income-well-being gradients and rescaled lottery estimates reported in the main text.

The methodology follows the following four general principles (outlined in the Analysis Plan). First, all else equal, gradients should be estimated in Swedish samples reweighted to match the sex and age distribution in the *Respondents Sample*. Second, all else equal, it is desirable to use outcomes defined as similarly as possible to the primary outcomes. Third, it is desirable to smooth out transitory fluctuations in year-to-year income whenever possible. And fourth, when multiple income measures are available, the measure most highly correlated with net annual household income is preferable.

The lottery estimates in eTable 11 are not on a scale that easily permits comparisons to income-health gradients. The lottery prizes we study represent substantial, one-time, increase in lifetime wealth. Previous work on the Swedish administrative sample has shown that large-prize winners enjoy sustained improvement in economic conditions that are robustly detectible for well over a decade after the windfall.[3–5] For example, winners reduce their labor supply following a win, but the reduction is modest, and persists for up two decades (and possibly longer; the number of lottery players who can be tracked for at least two decades is not yet large enough to allow well-powered analyses). The lottery wealth is spent down over time, but at a rate modest enough to detect associations between lottery wealth and financial assets and real estate wealth measured a decade after the lottery event. This evidence, which is consistent with the conclusions from interview-based research on lottery winners in multiple countries,[13–16] suggests lottery prizes induce a major shift in the long-run income status of the winner's household.

The general idea behind our comparison is therefore to measure, for each lottery prize, an approximate increase in annual income that it could sustain over a long time period. With lump-sum prizes converted to a measure of a long-run increase in annual income, our lottery estimates can be interpreted as associations with a permanent increase in annual income, and are more directly comparable to income well-being gradients. We have no unassailable method for translating the lump-sum prize to a corresponding increase, but following the Analysis Plan and previous research,[3] we proceed by translating each lottery prize into an annual income, measured as the annual payout the prize would generate if it were annuitized over a 20-year period at an actuarially fair return. For point of reference, a lump-sum prize of $100,000 translates into an increase in permanent annual after-tax income of $5,996.

Income Gradients in Respondents Sample and European Social Survey. In the main text, we compare rescaled lottery estimates with gradients estimated in the full *Respondents Sample*. To obtain a long-run measure of economic status purged of transitory year-to-year fluctuations in income, we defined the permanent annual income for each player as the average disposable household income over the period 2004-2014. Our measure of annual household income is net of taxes and we left censor annual observations at $6,000. We then estimate a well-being gradient for each primary outcome by regressing it on permanent income, controlling for sex, a fourth-order polynomial in age and sex-by-age interactions. A potential limitation of this approach is that some of the variation in our measure of permanent income is endogenous to the lottery. We therefore also ran these analyses in a sample restricted to small-prize winners, defined as players who won prizes smaller than $20K. In this sample, average prize won ($8,483) is small enough that any endogeneity is likely to be negligibly small. eTable 14 shows that gradients estimated using the full and restricted *Respondents Sample* are consistently very similar in magnitude.

We also compare the gradients in the *Respondents Sample* with gradients estimated among Swedish respondents in wave seven (2014) of the European Social Survey (ESS). To maximize comparability, we estimated the ESS gradients using the same sex and age controls, in a sample reweighted to match the sex and age distribution of the *Respondents Sample*. ESS respondents are asked to indicate their household income, net of taxes, by choosing one of several categories. Each category corresponds to an interval. We assign each respondent an income equal to the midpoint of the chosen interval. We set the annual after-tax income to 0.66M SEK (year-2014 prices) for

households in the top decile. For comparability, our final income variables are converted to units of year-2011 10K USD, and we apply the same left-censoring threshold ($6,000) as in the *Respondents Sample*.

For each of our primary outcomes, we sought to construct a similar variable in the ESS. Our ESS measures of subjective health, smoking and risk for alcohol dependency are derived from questions that are very similar to those used to construct the primary outcomes *Subjective Health*, *Smoking* and *Alcohol*. Our ESS analogue of the *Health Index* is a linear combination of 22 indicator variables derived from responses to the questions (1) "Which of the health problems on this card have you had or experienced in the past 12 months …", and "Which of the health problems on this card have you experience in the last 12 months hampered you in your daily activities in any way?". The item weights are derived from a regression analogous to the one used to construct *Health Index*. Our measure of physical activity is derived from responses to the question "On how many of the last 7 days did you walk quickly, do sports or other physical activity for 30 minutes or longer?" Finally, we use two questions from the ESS to construct a variable comparable to the primary outcome *Dietary Quality*. The first asks respondents how often they eat fruit and the second about vegetables (both measured on a 1-7 scale). The two scores are standardized and summed to form a single outcome variable.

eTable 14 compares gradients from our *Respondents Sample* to those in the ESS. In all specifications, the dependent variable has been standardized to have unit variance. For most outcomes, the gradients are similar in magnitude. For example, the estimated ESS and *Respondents Sample* gradients for *Subjective Health* are 0.080 (SE = 0.07) and 0.086 (SE = 0.012), respectively. Across the six outcomes, there is no systematic tendency for the gradients to be systematically steeper in either sample: for three out of the six primary outcomes, the absolute value of the estimated gradient is greater in the ESS. The ESS gradients in ESS are weaker for *Health Index* and *Physical Activity*, but these outcomes differ the most from our survey. In the ESS, the positive relationship between *Alcohol* and household income is stronger in the ESS than in the *Respondents Sample*, despite the two outcomes being measured similarly.

Comparison to Income Gradients. Having established that gradients in our restricted *Respondents Sample* replicate standard patterns in the literature, we compare our rescaled lottery estimates to gradients from the *Respondents Sample*. The results from this comparison is shown in eTable 15 and Figure 4.

Comparison to Published Estimates from Lottery Studies. The Analysis Plan briefly mentioned our intention to benchmark our findings against estimates in previous quasi-experimental studies, especially studies of lottery players. Here, we describe the inclusion criteria used to identify the final list of quasi-experimental estimates used in our final comparisons. We also explain how we transformed the estimates in the original studies to make them comparable to ours.

We conducted a systematic search for quasi-experimental studies of lottery players' overall health or health behaviors. We identified four studies that analyzed at least one outcome comparable to one of our six primary outcomes.[8,17–19] However, two of these analyzed a very similar set of outcomes in the same dataset (the British Household Panel Survey).[17,19] To simplify the exposition, we only retained one of them.

The first study used data from three waves of SLLS to study the health of Swedish lottery winners.[8] The SLLS survey data can be used to calculate the sum of monetary prizes won between 1969 and 1981. Winners are not asked about the exact year of win or the name of the lottery, however. The study therefore compared the health outcomes of N = 626 winners who won prizes of different magnitude – the key identifying assumption for causal inference is that in this sample of winners, the reported lottery wealth is as good as randomly assigned. One of the health outcomes, an index of *Bad Health*, closely resembles our *Health Index* (except that it is coded so greater values denote worse health). Table 3 of the study reports that the estimated effect of 130,000 SEK (year-1998 prices) on *Bad Health*, measured up to 12 years after the win, ranges from -0.04 to -0.07 SD units. The estimates vary depending on the set of controls used (but the standard error, rounded to three decimal places, is 0.029 in all three specifications). We therefore use the midpoint of these estimates, -0.055, in our comparison. To make this estimate comparable to ours, we first calculated that 130,000 year-1998 SEK is equal to 22,264 year-2011 USD. The estimates must therefore be converted by a factor of -100,000/22,264 = -4.49. The negative sign is needed to align the directional coding of the two indices. Applying this conversion factor gives a rescaled estimates of 0.25 SD units (SE = 0.13) to be compared to our estimated lottery estimate for *Health Index*, which is equal to -0.003 (SE = 0.015).

Raschke[18] uses the German Socioeconomic Panel to study the effects of lottery wealth on a number of outcomes comparable to ours. Using the longitudinal data, he analyzes within-person changes in the outcome shortly after a large win (2,500 euros or more). The study's key identifying assumption is therefore that, in the sample of large-prize winners, the timing of the win is unrelated to other time-varying and unobserved factors that affect the outcome. In his primary specification, used in our comparisons below, Raschke estimates the association with an indicator variable for having won a large prize in the last year.

The study considers three measures of overall health. The first is an index of physical health which we consider comparable to our *Health Index*. The next two variables are binary and comparable to the question about self-assessed overall health we used to generate *Subjective Health*. Raschke[18] defines *Bad Health* as an indicator variable equal to one for individuals who indicate that their overall health is "Poor" or "Very Poor". His second variable – *Good Health* – is an indicator variable for individuals who rate their health as "Good" or "Very Good". The study also analyzes binary variables for smoking and frequent drinking (but not quantities consumed).

The study reports a small and statistically insignificant negative association with the index of physical health. In SD units, the estimate is -0.01 (SE = 0.086). To facilitate comparisons to the four binary outcomes, we constructed similar outcomes in our sample and reran our main analyses. Specifically, we use our question about self-assessed health and the same cutoffs as Raschke to generate binary indicators for *Good Health* and *Bad Health.* We classify a person as a smoker if he or she reports a daily consumption of cigarettes greater than zero. To get a binary measure of drinking, we define an indicator variable for respondents whose score on our screening test exceeded 7, the cutoff for dependence recommended by a recent validation study conducted in Sweden.[20] Raschke finds that immediately following a win, winners are 7.7 percentage points (SE = 2.4) more likely to evaluate their own health status as bad or very bad (*Bad Health*), 4.6 percentage points (SE = 2.9) less likely to evaluate their own health as good or very good (*Good Health*), 2.4 percentage points (SE = 2.4) less likely to be smokers and 3.3 percentage points (SE = 3.3) less likely to drink alcohol frequently.

From Raschke's Table 1, we calculate that the average large prize won by players in his sample was 24,249 EUR in year-2005 prices. In year-2011 USD, the average prize is 45,078 USD. Therefore, Rischke's estimates need to be rescaled by a factor of 100,000/45,078=2.22.

Raschke's rescaled estimates imply that a net wealth shock of $100,000 is associated with 0.02 (SE = 0.19) lower index of physical health, 17.1 percentage points (SE = 5.4) lower probability of being in *Bad Health*, 10.1 percentage points (SE = 6.3) lower probability of being in *Good Health*, 5.4 percentage points (SE = 5.2) lower probability of being a smoker, and 7.4 percentage points (SE = 7.3) lower probability of drinking alcohol regularly. Our lottery estimates for all four binary outcomes are close to zero, with standard errors at least eight times smaller. For example, estimates based on our sample suggest that a net wealth shock of $100,000 reduces the long-run probability of *Bad Health* by less than one tenth of a percentage point, compared to 17.1 percentage points (SE = 5.4).

Three out of the four comparisons of binary outcomes are based on outcomes that were defined using very similar procedures in the two studies. For *Alcohol*, an interpretational caveat is that our binary variable is derived from a score on a test designed to screen individuals at risk for alcohol dependence. Alcohol dependence is conceptually distinct from frequent consumption of alcohol. Since the two variables are empirically related, we find the comparison informative nonetheless, but we urge caution in making this specific comparison.

A study based on the British Household Panel Study concludes that lottery wealth is associated with more smoking and social drinking in the years following a win.[17,19] For smoking, this conclusion is based in part on a comparison of the outcomes of "big-prize winners" (defined as winners who report prizes greater than £500 in year-2005 prices) to small-prize winners in (i) the two years following a win (ii) the year following a win or (iii) the year-of-win. For expositional ease, our comparisons below are restricted to estimates based on the two-year comparisons of big-prize and small-prize winners. However, none of our conclusions change substantively if we instead use estimates that are based on tighter follow-up windows, or one of the other identification strategies used in the study.

The study reports results for binary and non-binary measures of smoking and drinking (specifically, an index of social drinking). Our primary outcomes *Alcohol* and *Cigarettes* are not binary, so we use the variables measuring the quantities consumed in our comparisons. Relative to the small-prize winners, the study finds that big-prize winners smoke 0.936 more cigarettes per day (SE = 0.341) and score 0.035 points higher on an index of drinking (SE = 0.041). Using frequencies depicted in the paper's Figure 1, we infer that the standard deviation of the index is approximately 1.5. In SD units the difference in the drinking index is therefore 0.023 (SE = 0.028). The study also reports that big-prize winners are 0.99 percentage points (SE = 1.19) less likely to report that their self-assessed health is in the highest category of a five-point response scale. This is the only measure of overall health analyzed, so we use it in our comparisons.

The study reports the average size of small prizes (£61.64, see p. 525), the fraction of prizes that are big wins (6%, see p. 524) and the average prize size overall (£245, see p. 524). From this information, we infer that the average big prize is approximately £3,120 in year-2005 prices, or $5,800 in year-2011 prices. Hence, we multiply the estimates by 100,000/5,800 = 17.24.

After rescaling, the estimated association is therefore 16 cigarettes (SE = 5.88), or 1.872 (SE = 0.682) in SD units. By contrast, our standardized lottery estimate is -0.006 (SE = 0.016). For the drinking index, the rescaled estimate

© 2020 Östling R et al. *JAMA Network Open.*

is instead 0.40 SD units (SE = 0.48), compared to our estimate of 0.003 SD units (SE = 0.015). Finally, the rescaled estimate implies a 17-percentage point reduction (SE = 20.5) in the probability of reporting the highest health category. For a similarly defined outcome in our sample, the corresponding estimate is 0.90 percentage points (SE = 0.70).

All three previous studies conclude that lottery wealth impacts at least one of our primary outcomes. One way to reconcile our overall pattern of null results with the findings in these previous studies is to argue that short-run effects of wealth are substantially larger than long-run effects. This explanation may have some merit, but it fails to explain some patterns in the data. A first is that Lindahl found large positive associations with a long-run measure of overall health. A second is that Apouey and Clark's results do not suggest a systematic tendency for the lottery wealth to have a large effect in the year-of-win that subsequently decays. A second possibility is that the previous studies relied on identification strategies that required stronger identifying assumptions for causal inference. Indeed, none of the previous studies compared the outcomes of players from the same lottery, because the data sets used do not contain information about the lottery associated with each prize won.

Under assumptions about realistic effect sizes that are informed by our new evidence, it is plausible that the earlier studies were underpowered. To illustrate some of the consequences of low power, suppose the true treatment effect on *Health Index* is smaller than 0.033 SD units (the upper limit of the 95% confidence interval of our estimate). The statistical power to detect an effect, at $\alpha = 0.05$, of such magnitude did not exceed 5.7% in any of the above three studies. Conditional on finding a statistically significant effect, design calculations[21] reveal that studies with statistical power this low will report an estimated effect with the wrong sign ("type S error") between 23% and 42% of the time. Moreover, the expected overestimate of the true effect size ("type M error") ranges from 9 to 34.

© 2020 Östling R et al. *JAMA Network Open.*

eReferences

1 Lindqvist E, Östling R, Cesarini D. Long-run Effects of Lottery Wealth on Psychological Well-being. *Rev Econ Stud.* Forthcoming.

2 Östling R, Lindqvist E, Cesarini D. The Effect of Wealth on Survey Outcomes. Analysis Plan Archived on the Open Science Framework on November 23, 2016. Available at https://osf.io/t3qb5/. .

3 Cesarini D, Lindqvist E, Östling R, Wallace B. Wealth, Health, and Child Development: Evidence from Administrative Data on Swedish Lottery Players. *Q J Econ* 2016; **131**: 687–738.

4 Briggs J, Cesarini D, Lindqvist E, Östling R. Windfall Gains and Stock Market Participation. *NBER Work Pap No 21673* 2015.

5 Cesarini D, Lindqvist E, Notowidigdo MJ, Östling R. The Effect of Wealth on Individual and Household Labor Supply: Evidence from Swedish Lotteries. *Am Econ Rev* 2017; **107**: 3917–46.

6 Herzog AR, Rodgers WL. Age and Response Rates to Inteview Sample Surveys. *J Gerontol* 1988; **43**: S200–5.

7 Jonsson JO, Mills C. The Swedish Level of Living Surveys: A General Overview and Description of the Event History Data. In: Cradle to Grave. Life-Course Change in Modern Sweden. Durham: Sociology Press, 2001: 228–42.

8 Lindahl M. Estimating the effect of income on health and mortality using lottery prizes as an exogenous source of variation in income. *J Hum Resour* 2005; **40**: 144–168.

9 Bush K, Kivlahan DR, McDonell MB, *et al.* The AUDIT Alcohol Consumption Questions (AUDIT-C). An Effective Brief Screening Test for Problem Drinking</subtitle> *Arch Intern Med* 1998; **158**: 1789.

10 Craig CL, Marshall AL, Sjöström M, *et al.* International physical activity questionnaire: 12-Country reliability and validity. *Med Sci Sports Exerc* 2003; **35**: 1381–95.

11 Zeger S, Liang K. Longitudinal data analysis for discrete and continuous outcomes. *Biometrics* 1986; **42**: 121–30.

12 Westfall PH, Young SS. Resampling-Based Multiple Testing: Examples and Methods for P-value Adjustment. New York: Wiley, 1993.

13 Kaplan HR. Lottery Winners: The Myth and Reality. *J Gambl Behav* 1987; **3**: 168–78.

14 Furåker, Bengt and Hedenus A. Gambling Windfall Decisions: Lottery Winners and Employment Behavior Journal. *UNLV Gaming Res Rev* 2009; **13**: 1–15.

© 2020 Östling R et al. *JAMA Network Open.*

15 Fleischer Eckblad G, von der Lippe AL. Norwegian Lottery Winners: Cautious Realists. *J Gambl Stud* 1994; **10**: 305–22.

16 Larsson B. Becoming a Winner But Staying the Same: Identities and Consumption of Lottery Winners. *Am J Econ Sociol* 2011; **70**: 187–209.

17 Apouey B, Clark AE. Winning big but feeling no better? The effect of lottery prizes on physical and mental health. *Health Econ* 2015; **24**: 516–38.

18 Raschke C. Unexpected windfalls, education, and mental health: evidence from lottery winners in Germany. *Appl Econ* 2019; **51**: 207–18.

19 van Kippersluis H, Galama TJ. Wealth and health behavior: Testing the concept of a health cost. *Eur Econ Rev* 2014; **72**: 197–220.

20 Lundin A, Hallgren M, Balliu N, Forsell Y. The Use of Alcohol Use Disorders Identification Test (AUDIT) in Detecting Alcohol Use Disorder and Risk Drinking in the General Population: Validation of AUDIT Using Schedules for Clinical Assessment in Neuropsychiatry. *Alcohol Clin Exp Res* 2015; **39**: 158–65.

21 Gelman A, Carlin J. Beyond Power Calculations: Assessing Type S (Sign) and Type M (Magnitude) Errors. *Perspect Psychol Sci* 2014; **9**: 641–51.

© 2020 Östling R et al. *JAMA Network Open.*

eTable 1. Selecting Sample of Survey Respondents

	Kombi	Triss-Lumpsum	Triss-Monthly	Total
	(1)	(2)	(3)	(4)
Time Period	1998-2011	1994-2011	1997-2011	1994-2011
# Prizes Awarded	499	5,057	824	6,380
<u>Original Restrictions</u>				
# Quality Control	7	190	36	233
# Shared Prize	0	342	61	403
# Multiple Winners in Cell	0	8	0	8
# Age <18 at Win	0	19	0	19
# Born <1941	230	1203	119	1552
# <4 Valid Controls (Kombi)	3	0	0	3
# Deceased before 2011	0	1	0	1
<u>Statistics Sweden</u>				
# Deceased, Emigrated, No Address	18	229	38	285
<u>Survey Population</u>				
# Prizes	241	3065	570	3876
# Controls	964	0	0	964
N	**1,205**	**3,065**	**570**	**4,840**
# Unique Individuals	1,196	3,061	570	4,820
<u>Survey Respondents</u>				
Survey Repondents	909	1,977	365	3,251
Abbreviated Survey	20	78	13	111
N	**929**	**2,055**	**378**	**3,362**
# Unique Individuals	**920**	**2,051**	**378**	**3,344**

This table summarizes the sample restrictions used to generate the *Survey Population* and shows the number of respondents to the survey. Failed quality control includes winners without ticket information (Kombi only), missing or incorrect PIN, uncertainty about the identity of the winner, missing information about shared prizes etc.

© 2020 Östling R et al. *JAMA Network Open.*

eTable 2. Distribution of Prizes Awarded

	Survey Population				Respondents Sample			
			Triss…				Triss…	
	All	Kombi	Lumpsum	Monthly	All	Kombi	Lumpsum	Monthly
	(1)	(2)	(3)	(4)	(5)	(6)	(7)	(8)
0	964	964	0	0	747	747	0	0
5K to 10K	811	0	811	0	554	0	554	0
10K to 50K	1896	0	1896	0	1261	0	1261	0
50K to 100K	211	0	211	0	138	0	138	0
100K to 200K	340	213	42	85	247	163	27	57
200K to 400 K	322	21	43	258	216	14	34	168
400K to 600K	149	4	26	119	104	4	18	82
600K to 800K	83	2	18	63	55	0	12	43
800K to 1M	52	0	18	34	32	0	11	21
>1M	12	1	0	11	8	1	0	7
Prize Sum (M)	410.7	44.4	128.3	237.9	277.2	33.3	86.1	157.8
% of Survey Pop.					67%	75%	67%	66%
N	4,840	1,205	3,065	570	3,362	929	2,055	378
% of Survey Pop.					69%	77%	67%	66%

This table compares the distribution of prizes in the *Respondents Sample* and in the *Survey Population* from which it was sampled. All prizes are after tax and measured in year-2011 USD. Triss-Monthly prizes have been converted to net present value assuming a 2% discount rate. The average monthly installment in the *Respondents Sample* was $2,547 and was on average paid out over 15.9 years.

eTable 3. Administrative Variables and Baseline Controls

**Panel A. Principal Administrative Variables**			
Basic Demographics (1990-)			
Sex	Civil Status	Educational Attainment	Birth Year
Immigrant	Children in Household		
Annual Income (1990-)			
Capital Income	Labor Income	Disposable Income	
Year-End Wealth (1999-2007)			
Total Assets	Total Debt	Net Wealth	

© 2020 Östling R et al. *JAMA Network Open.*

Panel B. Definition of Baseline Controls				
1. Age	2. Age^2	3. 1 if Female	4. 1 if College	5. 1 if Married
5. 1 if Born in Sweden		6. No. Children	7. Capital Income	8. Labor Income

Panel A lists key administrative variables from Statistics Sweden the survey population has been matched to. Panel B defines the set of baseline controls that are included as covariates in the main estimating equation and in our tests of covariate balance. To reduce the influence of outliers, we winsorize all wealth and income variables at the 0.5th and 99.5th percentile prior to analyses. All monetary variables are measured in year-2011 USD. Age is measured in the year of the lottery event. #Children is number of children (below 18) domiciled in the respondent's household. Income assumed to be zero in five instances when information is unavailable, but is dropped when income is used as an outcome variable.

© 2020 Östling R et al. *JAMA Network Open.*

eTable 4. Representativeness of Survey Respondents

	Respondents Sample				Survey Population	Weighted Representative Sample
	Kombi	Triss-Lumpsum	Triss-Monthly	Pooled		
	(1)	(2)	(3)	(4)	(5)	(6)
Year of Birth	1951	1957	1958	1956	1957	1956
S.D.	8	12	12	11	12	11
1 if Female	40%	52%	49%	48%	47%	48%
1 if College	24%	26%	28%	26%	22%	30%
1 if Swedish-born	95%	91%	92%	92%	91%	84%
1 if Married	53%	54%	54%	54%	48%	51%
# Children	0.33	0.69	0.62	0.58	0.62	0.56
S.D.	0.73	1. 0	0.94	0.94	0.97	0.95
Annual Capital Income (USD)	-626	-979	-691	-849	-964	-26
S.D.	5,413	7,871	7,463	7,226	6,707	8,465
Annual Labor Income (USD)	37,455	33,431	37,160	34,963	33,875	32,075
S.D.	22,598	21,748	22,277	22,123	21,894	24,671
N	929	2,055	378	3,362	4,840	373,276

Column (1) to (4) show mean values of the baseline controls for the different lotteries in the *Respondents Sample*. Column (5) shows the corresponding data for the *Survey Population* and column (6) shows a representative sample from 2010 weighted by the sex and age distribution in the *Respondents Sample*. All time-varying variables are measured the year prior to the lottery event.

© 2020 Östling R et al. *JAMA Network Open.*

eTable 5. Prevalence in Respondents Sample vs Swedish Level of Living Survey

	Respondents Sample	SLLS 2010			Respondents Sample	SLLS 2010
**Pain**				**Stomach/intestinal Problems**		
Pain in shoulders	38%	29%		Stomach pains	13%	13%
Pain in the joints	44%	25%		Constipation	6%	5%
Pain in the back	44%	35%		Gastric ulcer	1%	2%
				Hemorrhoids, rectal pain	9%	5%
**Cardiovascular Diseases**				Nausea	6%	5%
Pain in chest	8%	6%				
Heart attack, coronary	1%	1%		**Other**		
Heart weakness	2%	3%		Weight loss	2%	2%
Stroke	1%	2%		Impaired vision not improved by glasses	4%	10%
High blood pressure	26%	24%		Hearing impairment	16%	18%
Varicose vein/ulcer	6%	5%		Chronic bronchitis/asthma	5%	5%
Swollen legs	10%	8%		Bladder/Prostate	6%	5%
Shortness of breath	8%	6%		Struma	2%	3%
Dizziness	13%	10%		Kindey problem/stone	2%	2%
				Overstrained	5%	3%
**Poor Mental Health**				Hot flushes (sweating)	11%	8%
General tiredness	27%	17%		Cancer	2%	2%
Sleeping problems	25%	20%		Diabetes	11%	7%
Nervousness/anxiety	13%	9%		Pains (injury or accident)	7%	7%
Depression	5%	6%				
Mental illness	1%	1%		**Overweight**		
				Overweight	18%	12%

This table reports the prevalence of the 35 health conditions included in the survey in the *Respondents Sample* and the 2010 wave of the Swedish Level of Living Survey (SLLS 2010). The prevalence reported for SLLS is calculated after reweighting of the sample to match the sex and age distribution in the *Respondents Sample*. Most variables are derived from questions that were not formulated identically. Moreover, the SLLS data are based on face-to-face interviews rather than a mail-in survey.

© 2020 Östling R et al. *JAMA Network Open.*

eTable 6. Health and Habits in Respondents Sample vs Representative Survey

	**Respondents Sample**		**Public Health Survey 2016**
Very good or good health	69%		68%
Very bad or bad health	6%		6%
At least 5 hours physical activity per week	26%		34%
Eat seafood weekly	72%		81%
Consume sweet drinks weekly	36%		34%
Smoke daily	11%		10%
Never drink alcohol	11%		14%
Risky alcohol consumption	21%		14%

This table compares responses in the *Respondents Sample* to the representative Swedish Public Health Survey 2016 (N = 9,219 to N = 9,371). The survey questions and the construction of the outcomes above were identical in the two surveys. Because we did not have access to complete data from the Swedish Public Health Survey, we can only do the comparison for a selected set of publicly available binary outcomes. Moreover, we could only re-weight the data from the representative survey to match the *Respondents Sample* based on sex and four age categories.

© 2020 Östling R et al. *JAMA Network Open.*

eTable 7. Summary Overview of Primary Outcomes

Subjective Health	Subjective evaluation of overall health					
Health Index	Weighted average of 35 health conditions (an ailment, disease or symptom)					
Cigarettes	Number of cigarettes smoked per day.					
Alcohol	Score on screening test for problem drinking.					
Physical Activity	Weekly energy expenditure (metabolic-equivalent minutes) on exercise, walking and biking.					
Dietary Quality	Index of dietary quality based on consumption of sweet drinks, seafood and vegetables.					
	**Pairwise Correlations**					
	Subjective Health	Health Index	Cigarettes	Alcohol	Physical Activity	Dietary Quality
Subjective Health	1.00	0.58	-0.11	0.04	0.29	0.13
Health Index	0.58	1.00	-0.08	0.09	0.19	0.04
Cigarettes	-0.11	-0.08	1.00	0.08	-0.13	-0.11
Alcohol	0.04	0.09	0.08	1.00	-0.01	-0.02
Physical Activity	0.29	0.19	-0.13	-0.01	1.00	0.19
Dietary Quality	0.13	0.04	-0.11	-0.02	0.19	1.00

© 2020 Östling R et al. *JAMA Network Open.*

eTable 8. Testing Endogenous Selection into the Respondent Sample

Outcome	**Returned Original Survey**		**Returned Abbreviated Survey**	**Returned Either Survey**
Sample	Survey Population		Follow-up Sample	Survey Population
	(1)	(2)	(3)	(4)
Lottery Wealth ($100K)	-0.0057	-0.0024	0.0077	-0.0024
SE	(0.0040)	(0.0059)	(0.0183)	(0.0058)
p (analytical)	[0.154]	[0.677]	[0.675]	[0.682]
p (resampling)	[0.153]	[0.666]	[0.634]	[0.681]
N	4,840	4,840	501	4,840
Proportion	67.2%	67.2%	22.2%	69.5%
Group FEs	No	Yes	Yes	Yes

This table reports the results from regressing a dummy variable indicating whether the survey was completed on the amount won (*Diagnostic Test 1*). Due to ethical concerns, we are not allowed to combine information about who responded to the survey with administrative variables, and these regressions do therefore not include any of the baseline control variables.

© 2020 Östling R et al. *JAMA Network Open.*

eTable 9. Testing for Conditional Random Assignment of Lottery Prizes

	**Survey Population**					**Respondents Sample**			
Kombi	X	X	X			X	X	X	
Triss-Monthly	X	X		X		X	X		X
Triss-Lumpsum	X	X		X		X	X		X
	(1)	(2)	(3)	(4)		(5)	(6)	(7)	(8)
Fixed Effects	None	Group ID	Group ID	Group ID		None	Group ID	Group ID	Group ID
N	4,840	4,840	1,205	3,635		3,362	3,362	929	2,433
Pre-Lottery Characteristics									
Age (Beta/SE)	0.525	1.049	N/A	1.045		0.274	0.798	N/A	0.709
p (analytical)	0.599	0.294	N/A	0.296		0.784	0.425	N/A	0.478
Age^2 (Beta/SE)	-0.710	-0.782	N/A	-0.809		-0.366	-0.550	N/A	-0.485
p (analytical)	0.478	0.435	N/A	0.419		0.714	0.582	N/A	0.628
1 if Female (Beta/SE)	0.952	0.792	N/A	0.809		1.006	0.959	N/A	1.002
p (analytical)	0.341	0.429	N/A	0.418		0.314	0.338	N/A	0.317
1 if College (Beta/SE)	0.750	1.516	-0.278	1.732		1.150	1.508	0.086	1.619
p (analytical)	0.453	0.130	0.781	0.083		0.250	0.132	0.932	0.106
1 if Married (Beta/SE)	0.118	-0.594	-0.971	-0.290		0.127	-0.769	-1.375	-0.303
p (analytical)	0.906	0.552	0.332	0.772		0.899	0.442	0.169	0.762
1 if Swedish (Beta/SE)	-1.197	-1.060	-1.091	-0.844		-1.497	-1.318	-1.503	-1.028
p (analytical)	0.231	0.289	0.275	0.399		0.135	0.187	0.133	0.304
#Children (Beta/SE)	-0.080	0.836	1.552	0.437		0.297	-0.049	0.599	-0.210
p (analytical)	0.936	0.403	0.121	0.662		0.766	0.961	0.549	0.833
Capital Income (Beta/SE)	0.098	-0.043	-1.609	0.157		-0.290	-0.593	-1.649	-0.446
p (analytical)	0.922	0.965	0.108	0.876		0.772	0.553	0.100	0.656
Labor Income (Beta/SE)	0.839	0.382	-0.314	0.477		1.199	0.652	-0.244	0.748
p (analytical)	0.402	0.702	0.754	0.633		0.230	0.514	0.808	0.455
Joint Test of Baseline Covariates									
F-statistic	0.716	1.247	1.054	1.262		0.889	1.256	1.021	1.265
p (analytical)	0.694	0.261	0.389	0.253		0.535	0.256	0.410	0.251
p (resampling)	0.629	0.317	0.371	0.314		0.352	0.225	0.408	0.314

© 2020 Östling R et al. *JAMA Network Open.*

This table reports results from our randomization tests in the *Survey Population* and the *Respondents Sample* (*Diagnostic Test 2*). Each column corresponds to a regression where the dependent variable is lottery prize and we control for baseline characteristics measured the year before the lottery event in all specifications. Under the null hypothesis of conditional random assignment, variables determined before the lottery should not have any predictive power conditional on the cell fixed effects. The table shows *t* statistics, i.e. coefficients divided by their standard error. The resampling based *p*-values are obtained from the resampling distribution of covariate coefficients from 10,000 Monte Carlo simulations. In each simulation, we permute the prizes within each group.

© 2020 Östling R et al. *JAMA Network Open.*

eTable 10. Lottery Estimates in Survey Population and Respondent Sample

		***t*=0 Net Wealth**			***t*=0 Total Debt**			***t*=1 Capital Income**			***t*=1 Labor Income**	
		Survey Population	Respondents Sample		Survey Population	Respondents Sample		Survey Population	Respondents Sample		Survey Population	Respondents Sample
		(1)	(2)		(3)	(4)		(5)	(6)		(7)	(8)
Lottery Wealth ($100)		53.256	53.523		-2.392	-1.178		0.722	0.532		-1.196	-1.211
SE		(3.836)	(5.110)		(0.978)	(1.500)		(0.197)	(0.178)		(0.180)	(0.219)
p (analytical)		[<0.001]	[<0.001]		[0.015]	[0.433]		[<0.001]	[0.003]		[<0.001]	[<0.001]
p (resampling)		[<0.001]	[<0.001]		[0.001]	[0.180]		[<0.001]	[0.003]		[<0.001]	[<0.001]
Mean		84,637	90,951		43,387	44,482		-331	-195		32,857	33,966
S.D.		137,053	141,577		54,072	53,325		8,329	8,918		22,677	23,175
N		1,976	1,403		1,976	1,403		4,129	2,901		4,129	2,901
Year-of-Win Restrictions		2000-2007			2000-2007			1994-2009			1994-2009	

This table compares the relationship between lottery winnings and income and wealth in the *Survey Population* to the corresponding estimate in the *Respondents Sample* (*Diagnostic Test 3*). All specifications include controls for the baseline characteristics measured at *t* = -1 and the lag of the dependent variable. Triss-Monthly is exlcuded in columns (1)-(4). The sample restrictions in (1)-(4) are needed because wealth data are only available 1999-2007. The restrictions in (5)-(8) are needed because income and capital income data are not available for the *Survey Population* after 2010.

© 2020 Östling R et al. *JAMA Network Open.*

eTable 11. Subjective Health and Health Behaviors (Primary Outcomes)

	Subjective Health	Health Index		Cigarettes	Alcohol	Physical Activity	Dietary Quality
	(1)	(2)		(3)	(4)	(5)	(6)
**Descriptive Statistics**							
Min	1.00	-0.54		0.00	0.00	0.00	-7.30
Max	5.00	2.04		40.00	12.00	2,580	5.36
Mean	3.83	1.60		1.41	3.21	1,153	0.00
SD	0.83	0.32		4.38	2.25	728	2.01
**Unstandardized Estimates**							
Lottery Wealth ($100K)	0.011	-0.001		-0.026	0.006	1.059	-0.014
SE	(0.013)	(0.005)		(0.072)	(0.034)	(11.422)	(0.034)
p (analytical)	[0.386]	[0.831]		[0.721]	[0.860]	[0.926]	[0.686]
p (resampling)	[0.375]	[0.822]		[0.685]	[0.856]	[0.923]	[0.644]
**Standardized Estimates**							
Lottery Wealth ($100K)	0.013	-0.003		-0.006	0.003	0.001	-0.007
SE	(0.015)	(0.015)		(0.016)	(0.015)	(0.016)	(0.017)
p (analytical)	[0.386]	[0.831]		[0.721]	[0.860]	[0.926]	[0.686]
p (resampling)	[0.369]	[0.828]		[0.693]	[0.849]	[0.930]	[0.655]
FWER *p*	[0.927]	[0.927]		[0.927]	[0.927]	[0.927]	[0.927]
N	3,338	3,158		3,204	3,087	3,061	3,246

This table shows the relationship between winning 100K USD and the six primary outcomes measured in standard-deviation units. Baseline controls and cell fixed effects are included in all regressions. Standard errors are clustered at the level of the individual. The resampling based *p*-values are obtained from the resampling distribution of the test statistic from 100,000 Monte Carlo simulations, permuting the prizes within each cell in each permutation. The family-wise error rate (FWER) is calculated using the free step-down resampling method of Westfall and Young (1993).

© 2020 Östling R et al. *JAMA Network Open.*

eTable 12. Robustness Analyses

	Subjective Health	Health Index		Cigarettes	Alcohol	Physical Activity	Dietary Quality
	(1)	(2)		(3)	(4)	(5)	(6)
**Original Estimate**							
Lottery Wealth ($100K)	0.013	-0.003		-0.006	0.003	0.001	-0.007
SE	(0.015)	(0.015)		(0.016)	(0.015)	(0.016)	(0.017)
**Reweighted Estimate**							
Lottery Wealth ($100K)	0.032						
SE	(0.019)						
p (analytical)	[0.094]						
p (resampling)	[0.260]						
N	3,338						
**Drop Large Prizes (above 500K USD)**							
Lottery Wealth ($100K)	-0.018	-0.040		-0.001	-0.002	0.061	-0.007
SE	(0.028)	(0.032)		(0.024)	(0.026)	(0.030)	(0.028)
p (analytical)	[0.525]	[0.218]		[0.956]	[0.938]	[0.045]	[0.792]
p (resampling)	[0.545]	[0.186]		[0.963]	[0.942]	[0.045]	[0.799]
N	3,194	3,019		3,067	2,951	2,928	3,105
**Drop Large Prizes (above 4M SEK)**							
Lottery Wealth ($100K)	-0.006	-0.016		-0.011	0.012	0.059	-0.008
SE	(0.026)	(0.028)		(0.024)	(0.026)	(0.026)	(0.025)
p (analytical)	[0.819]	[0.558]		[0.661]	[0.637]	[0.026]	[0.749]
p (resampling)	[0.818]	[0.534]		[0.658]	[0.610]	[0.027]	[0.739]
N	3,237	3,061		3,110	2,994	2,969	3,148

This table reports the results from two robustness tests. The first robustness check reweight respondents to the abbreviated phone interview to match the population response rate to the mail-in survey, i.e. the abbreviated survey respondents are given a weight of 14.3. This robustness check is not feasible for financial life satisfaction and mental health because these outcomes were not measured in the abbreviated survey. The second robustness check reports the results when excluding very large prizes (above 500K USD and 4M SEK).

© 2020 Östling R et al. *JAMA Network Open.*

eTable 13. Heterogeneity Analyses

		Time since lottery		Lottery Type		Disp. Income		Age at time of win		Sex	
		Won Before 2005	Won 2005 or Later	Triss-Lumpsum	Triss-Monthly	Below Median	Above Median	Below Age 51	Age 51 or Older	Male	Female
		(1)	(2)	(3)	(4)	(5)	(6)	(7)	(8)	(9)	(10)
Subjective Health	Lottery Wealth ($100K)	0.046	-0.022	0.052	0.002	0.001	0.022	0.016	0.016	0.006	0.021
	SE	(0.020)	(0.023)	(0.022)	(0.023)	(0.022)	(0.016)	(0.019)	(0.019)	(0.018)	(0.019)
	p	[0.021]	[0.343]	[0.017]	[0.924]	[0.973]	[0.183]	[0.391]	[0.387]	[0.730]	[0.270]
	p equal	[0.026]		[0.111]		[0.352]		[0.994]		[0.498]	
	N	1,667	1,671	2,038	377	1,440	1,894	1,668	1,670	1,721	1,617
Health Index	Lottery Wealth ($100K)	0.022	-0.034	0.019	-0.017	-0.013	0.001	-0.005	0.001	0.005	-0.010
	SE	(0.021)	(0.022)	(0.023)	(0.021)	(0.022)	(0.016)	(0.020)	(0.018)	(0.015)	(0.021)
	p	[0.311]	[0.120]	[0.402]	[0.436]	[0.565]	[0.928]	[0.780]	[0.943]	[0.757]	[0.628]
	p equal	[0.069]		[0.253]		[0.523]		[0.759]		[0.479]	
	N	1,574	1,584	1,918	358	1,362	1,792	1,569	1,589	1,629	1,529
				.							
Cigarettes	Lottery Wealth ($100K)	-0.001	-0.011	0.001	-0.017	-0.029	0.009	0. 00	-0.010	-0.008	-0.002
	SE	(0.018)	(0.028)	(0.029)	(0.019)	(0.017)	(0.021)	(0.024)	(0.015)	(0.016)	(0.023)
	p	[0.954]	[0.687]	[0.985]	[0.353]	[0.087]	[0.674]	[0.995]	[0.532]	[0.615]	[0.918]
	p equal	[0.758]		[0.601]		[0.063]		[0.651]		[0.793]	
	N	1,596	1,608	1,952	359	1,371	1,829	1,595	1,609	1,650	1,554
				.							
Alcohol	Lottery Wealth ($100K)	0.009	-0.003	0.011	0.004	-0.016	0.015	0.028	-0.017	0.014	-0.009
	SE	(0.024)	(0.019)	(0.024)	(0.021)	(0.020)	(0.018)	(0.021)	(0.016)	(0.018)	(0.019)
	p	[0.708]	[0.874]	[0.630]	[0.865]	[0.421]	[0.415]	[0.173]	[0.293]	[0.454]	[0.618]
	p equal	[0.695]		[0.801]		[0.154]		[0.033]		[0.276]	
	N	1,536	1,551	1,888	348	1,324	1,759	1,553	1,534	1,596	1,491
				.							
Physical Activity	Lottery Wealth ($100K)	0.020	-0.019	0.015	-0.023	-0.003	0.006	0.027	-0.019	-0.013	0.016
	SE	(0.022)	(0.022)	(0.022)	(0.025)	(0.019)	(0.019)	(0.019)	(0.019)	(0.017)	(0.021)
	p	[0.365]	[0.391]	[0.504]	[0.354]	[0.868]	[0.761]	[0.167]	[0.317]	[0.430]	[0.445]
	p equal	[0.212]		[0.255]		[0.682]		[0.039]		[0.181]	
	N	1,521	1,540	1,860	349	1,297	1,760	1,568	1,493	1,594	1,467
Dietary Quality	Lottery Wealth ($100K)	-0.006	-0.010	0.021	-0.038	-0.018	-0.001	0.008	-0.021	-0.011	-0.007
	SE	(0.019)	(0.028)	(0.020)	(0.030)	(0.019)	(0.021)	(0.017)	(0.023)	(0.022)	(0.018)
	p	[0.738]	[0.730]	[0.287]	[0.204]	[0.339]	[0.966]	[0.626]	[0.355]	[0.612]	[0.711]
	p equal	[0.925]		[0.099]		[0.438]		[0.207]		[0.839]	
	N	1,617	1,629	1,976	365	1,397	1,844	1,607	1,639	1,676	1,570

This table reports from five heterogeneity analyses. Columns (1) and (2) show result separately for winners before or after Jan 1 2005. Column (3) and (4) show the results separately for Triss-Lumpsum and Triss-Monthly winners. Columns (5) and (6) display results separately for those above or below the median income in a representative sample. This analysis is based on individual disposable income (in the pre-lottery year) and the population median is calculated conditional on the respondent's sex and age category (18-27, 28-37,…, 68+) in the year prior to the win. Column (7) and (8) show the result for winners above or below the median age in the sample. Finally, columns (9) and (10) show the results separately for men and women. All regressions include the baseline control variables.

© 2020 Östling R et al. *JAMA Network Open.*

eTable 14. Gradients in Respondents Sample and European Social Survey

	Subjective Health			Health Index			Cigarettes		
	Respondents Sample		ESS	Respondents Sample		ESS	Respondents Sample		ESS
	Small prizes	Full	Wave 7	Small prizes	Full	Wave 7	Small prizes	Full	Wave 7
	(1)	(2)	(4)	(5)	(6)	(8)	(9)	(10)	(12)
Gradient (10K)	0.079	0.080	0.086	0.066	0.069	0.035	-0.054	-0.053	-0.073
SE	(0.009)	(0.007)	(0.012)	(0.009)	(0.007)	(0.011)	(0.009)	(0.007)	(0.014)
N	2,109	3,320	1,301	2,005	3,140	1,303	2,032	3,187	1,303
	**Alcohol**			**Physical Activity**			**Dietary Quality**		
	Respondents Sample		ESS	Respondents Sample		ESS	Respondents Sample		ESS
	Small prizes	Full	Wave 7	Small prizes	Full	Wave 7	Small-prizes	Full	Wave 7
	(13)	(14)	(16)	(13)	(14)	(16)	(13)	(14)	(16)
Gradient (10K)	0.029	0.025	0.088	0.044	0.055	0.026	0.063	0.063	0.063
SE	(0.009)	(0.007)	(0.012)	(0.009)	(0.007)	(0.012)	(0.009)	(0.007)	(0.011)
N	1,948	3,069	1,156	1,941	3,044	1,302	2,057	3,228	1,302

This table compares the income-health gradient in our sample of small-prize winners (<20K) and the full *Respondents Sample* to wave 7 (2014) of the Swedish data from the European Social Survey (ESS). All gradients are estimated controlling for sex, a fourth-order age polynomial and sex-by-age interactions. To maximize comparability, the ESS regressions are weighted to ensure a sex- and age distribution matching the *Respondents Sample.* In the *Respondents Sample*, we define income as the respondent's average annual household disposable income between 2004 and 2014, left-censored at $6K in year-2011 prices, whereas we use the self-reported household income from ESS. We constructed outcomes in ESS to be maximally similar to the primary outcomes used in our survey. The Subjective Health measures are near-identical to those used in our survey. The Health Index is a linear combination of 22 dummy variables representing responses to the question "Which of the health problems on this card have you had or experienced in the past 12 months …", and , and "Which of the health problems on this card have you experience in the last 12 months hampered you in your daily activities in any way?". Weights are derived by regressing the subjective health rating on these dummy variables. The smoking and alcohol questions are similar to our survey and the outcomes are defined in the same way as our primary outcomes. The measure of physical activity in ESS is determined by responses to the question "On how many of the last 7 days did you walk quickly, do sports or other physical activity for 30 minutes or longer?" Dietary quality is quantified using two questions in the ESS that measure respondents' frequency of eating fruit and frequency of eating vegetables on a 1-7 scale. These two scores are standardized and summed to form a single outcome variable.

© 2020 Östling R et al. *JAMA Network Open.*

eTable 15. Comparison to Permanent-Income Gradients

	Subjective Health		Health Index		Cigarettes	
	Lottery	Gradient	Lottery	Gradient	Lottery	Gradient
	(1)	(2)	(3)	(4)	(5)	(6)
Rescaled Estimate ($10K)	0.022	0.080	-0.005	0.069	-0.010	-0.053
SE	(0.025)	(0.007)	(0.026)	(0.007)	(0.027)	(0.007)
p equal	[0.027]		[0.004]		[0.130]	
N	3,338	3,320	3,158	3,140	3,204	3,187
	**Alcohol**		**Physical Activity**		**Dietary Quality**	
	Lottery	Gradient	Lottery	Gradient	Lottery	Gradient
	(7)	(8)	(9)	(10)	(11)	(12)
Rescaled Estimate ($10K)	0.005	0.025	0.002	0.055	-0.011	0.063
SE	(0.026)	(0.007)	(0.026)	(0.007)	(0.028)	(0.007)
p equal	[0.425]		[0.050]		[0.008]	
N	3,087	3,069	3,061	3,044	3,246	3,228

This table compares lottery estimates to income-health gradients estimated in the *Respondents Sample*. Lottery estimates have been rescaled assuming lottery prizes are annuitized over 20 years at a 2% real interest rate. Baseline controls and cell fixed effects are included when estimating effect sizes, whereas gradients are estimated controlling for sex, a fourth-order age polynomial and sex-by-age interactions. Gradients are estimated using the respondent's average annual household disposable income between 2004 and 2014, left-censored at $6K in year-2011 prices. "*p* equal" is the *p*-value obtained from a Wald test that the rescaled causal estimate is equal to the gradient estimated in the full sample.

© 2020 Östling R et al. *JAMA Network Open.*

eFigure 1. Schematic Overview of Timeline for Collection of Survey Data

All data were collected in 2016.

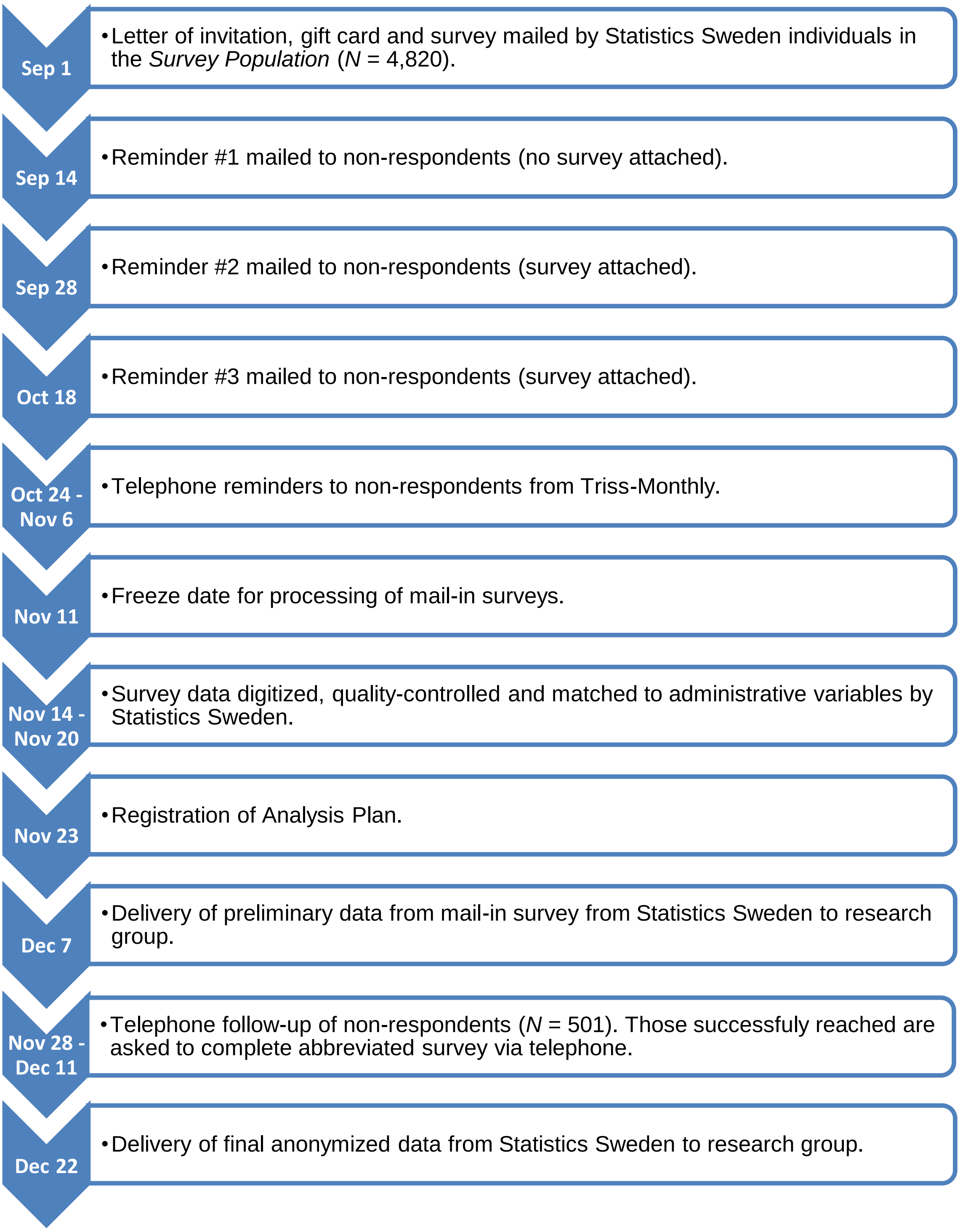

© 2020 Östling R et al. *JAMA Network Open.*

eFigure 2. Illustration of Identification Strategy in Kombi Sample

The original data are longitudinal and contain information about the universe of players who participated in monthly draws during over the course of a 12-year period. This figure illustrates how we generate the group in hypothetical data. We match each large-prize winner to four randomly chosen controls who owned the same number of tickets in the draw in question but did not win. To improve precision, we choose controls with the same age and sex as the winner.

No.	PIN	Winner	# tickets	Period
1	13	Yes	3	Dec-98
2	42	No	2	Dec-98
3	12	No	3	Dec-98
4	15	No	3	Dec-98
…				
99	76	No	3	Dec-98
100	45	No	1	Dec-98
101	14	Yes	4	Dec-98
102	34	No	1	Dec-98
103	16	No	1	Dec-98
104	765	No	4	Dec-98
…				
199	456	No	4	Dec-98
200	8	No	4	Dec-98
201	143	Yes	3	Jan-05
202	344	No	3	Jan-05
203	123	No	3	Jan-05
204	4	No	3	Jan-05
…				
299	656	No	3	Jan-05
300	7	No	3	Jan-05

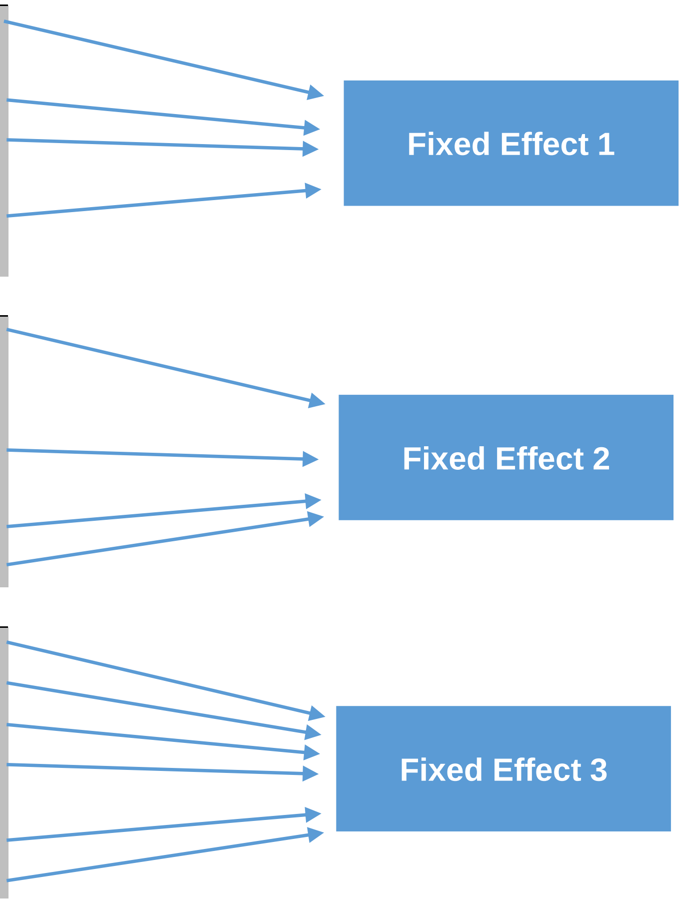

© 2020 Östling R et al. *JAMA Network Open.*

eFigure 3. Results from Pre-registered Robustness Analyses

In the first robustness analysis, we weight each abbreviated survey respondent such that the weighted fraction of mail-in survey respondents in the estimation sample matches the population fraction of 31%. In the second, we drop very large prizes (above 500K USD) from the estimation sample. The underlying data are in eTable 12.

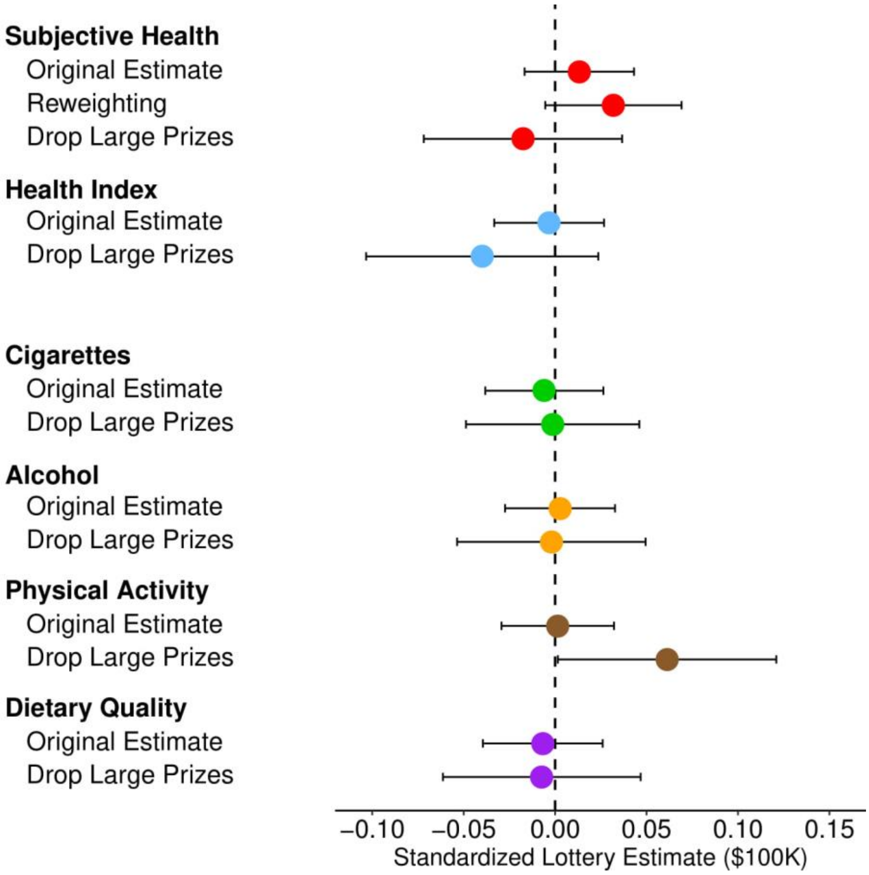

© 2020 Östling R et al. *JAMA Network Open.*

eFigure 4. Results from Pre-registered Heterogeneity Analyses

Each bar represents the lottery estimates for $100,000 USD (net of taxes) for outcomes measured in SD units. Lines denote 95% CIs. The underlying data are in eTable 13.

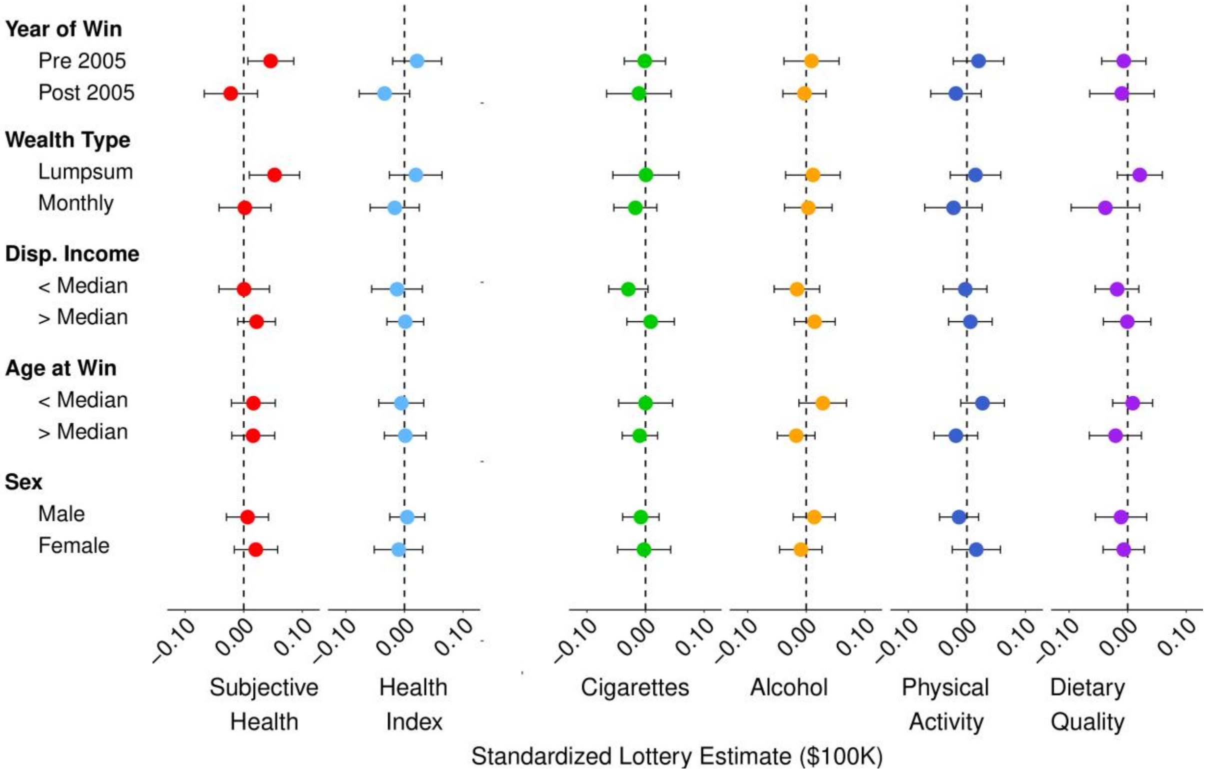

© 2020 Östling R et al. *JAMA Network Open.*
